# The zinc transporter ZIP12 regulates monocrotaline-induced proliferation and migration of pulmonary arterial smooth muscle cells via the AKT/ERK signaling pathways

**DOI:** 10.1186/s12890-022-01905-3

**Published:** 2022-03-28

**Authors:** Chaoyi Ye, Guili Lian, Tingjun Wang, Ai Chen, Weixiao Chen, Jin Gong, Li Luo, Huajun Wang, Liangdi Xie

**Affiliations:** 1 Department of Geriatrics, The First Affiliated Hospital of Fujian Medical University, 20 Chazhong Road, Fuzhou, 350005 Fujian People’s Republic of China; 2 Department of General Medicine, The First Affiliated Hospital of Fujian Medical University, Fuzhou, People’s Republic of China; 3 Fujian Hypertension Research Institute, The First Affiliated Hospital of Fujian Medical University, Fuzhou, People’s Republic of China

**Keywords:** ZIP12, Monocrotaline, Pulmonary arterial hypertension, Pulmonary arterial smooth muscle cells, Proliferation, Migration

## Abstract

**Background:**

The zinc transporter ZIP12 is a membrane-spanning protein that transports zinc ions into the cytoplasm from the extracellular space. Recent studies demonstrated that upregulation of ZIP12 is involved in elevation of cytosolic free zinc and excessive proliferation of pulmonary arterial smooth muscle cells (PASMCs) induced by hypoxia. However, the expression of ZIP12 and its role in pulmonary arterial hypertension (PAH) induced by monocrotaline (MCT) in rats have not been evaluated previously. The aim of this study was to investigate the effect of ZIP12 on the proliferation and migration of PASMCs and its underlying mechanisms in MCT-induced PAH.

**Methods:**

A PAH rat model was generated by intraperitoneal injection of 20 mg/kg MCT twice at one-week intervals. PASMCs were isolated from the pulmonary arteries of rats with MCT-induced PAH or control rats. The expression of ZIP12 and related molecules was detected in the lung tissues and cells. A ZIP12 knockdown lentivirus and an overexpressing lentivirus were constructed and transfected into PASMCs derived from PAH and control rats, respectively. EdU assays, wound healing assays and Western blotting were carried out to explore the function of ZIP12 in PASMCs.

**Results:**

Increased ZIP12 expression was observed in PASMCs derived from MCT-induced PAH rats. The proliferation and migration of PASMCs from PAH rats were significantly increased compared with those from control rats. These results were corroborated by Western blot analysis of PCNA and cyclin D1. All these effects were significantly reversed by silencing ZIP12. Comparatively, ZIP12 overexpression resulted in the opposite effects as shown in PASMCs from control rats. Furthermore, selective inhibition of AKT phosphorylation by LY294002 abolished the effect of ZIP12 overexpression on enhancing cell proliferation and migration and partially suppressed the increase in ERK1/2 phosphorylation induced by ZIP12 overexpression. However, inhibition of ERK activity by U0126 resulted in partial reversal of this effect and did not influence an increase in AKT phosphorylation induced by ZIP12 overexpression.

**Conclusions:**

ZIP12 is involved in MCT-induced pulmonary vascular remodeling and enhances the proliferation and migration of PASMCs. The mechanism of these effects was partially mediated by enhancing the AKT/ERK signaling pathways.

**Supplementary Information:**

The online version contains supplementary material available at 10.1186/s12890-022-01905-3.

## Introduction

Pulmonary arterial hypertension (PAH) is a fatal and serious disorder characterized by progressive pulmonary vascular remodeling, elevated pulmonary arterial pressure, leading to right heart failure related a poor prognosis [1]. The pathological mechanisms of PAH are quite complicated and involve intricate cellular and molecular processes such as endothelial dysfunction, vasoconstriction, in situ thrombosis, inflammatory cell infiltration, and vascular remodeling [2]. Pulmonary vascular remodeling occurs in all three layers of the vascular wall, including the endothelial cells, pulmonary arterial smooth muscle cells (PASMCs), and fibroblasts [3]. Currently, treatment strategies for PAH focus on vasodilation. Agents that target phosphodiesterase, prostacyclin, and endothelin-1 receptor pathways improve the quality of life and alleviate symptoms; however, disease progression cannot be radically prevented even by novel strategies based on multiple drug combinations [1, 4]. Previously, our group reported that pulmonary vascular remodeling occurs prior to manifestation of elevated pulmonary artery pressure in PAH models induced by monocrotaline (MCT), and this pathological process is accompanied by the elevation of pulmonary arterial pressure throughout the whole period of the disease [5]. However, no effective treatments are currently available to reverse pulmonary vascular remodeling.

Zinc is the second most abundant trace element after iron in all living organisms. Zinc is involved in a variety of biological processes, such as cellular metabolism, enzyme activity, and immune function [6]. Importantly, free zinc can serve as an intracellular second messenger in response to various external and internal stimuli [7]. It is well-known that zinc inhibits several protein tyrosine phosphatases and can thus activate various tyrosine kinase signaling pathways, including the PI3K/AKT and ERK signaling cascades [8–10]. Intracellular zinc homeostasis is tightly regulated by two families of zinc transporters, ZIP (Zrt/Irt-like protein, SLC39) and ZnT (zinc transporter, SLC30) [7]. The major function of ZIP channels is to increase the level of cytoplasmic zinc by mobilizing zinc from extracellular sources or intracellular stores, whereas ZnT transporters facilitate zinc transport in the reverse direction [7].

ZIP12 is a member of the ZIP family and localizes mainly on the cell membrane. The human ZIP12 gene is positioned in the short arm of chromosome 10 (10p12.33) and comprises 13 exons [11]. ZIP12 is most abundantly expressed in the central nervous system; hence, the studies on ZIP12 mainly focused on neurodevelopmental and psychiatric disorders [12–14]. ZIP12 contributes to an increase in zinc uptake and subsequent CREB phosphorylation, which is involved in tubulin polymerization and neurite elongation in neurons [12]. Upregulation of ZIP12 expression may contribute to the onset of schizophrenia by perturbing zinc homeostasis in the cerebral cortex [13]. In a recent study, it was proposed that ZIP12 is a key regulator of chronic hypoxia-induced pulmonary vascular remodeling [15]. ZIP12 knockdown was shown to inhibit hypoxia-induced influx of zinc ions and subsequent proliferation of the cells, ameliorate pulmonary arterial remodeling, and suppress elevated pulmonary arterial pressure [15]. Our previous study [16] and another recent study [17] have demonstrated that ZIP12 expression is upregulated in MCT-induced PAH. However, the role of ZIP12 in mechanisms of MCT-induced PAH rats have not been described previously.

Consequently, the present study aimed to determine whether ZIP12 is implicated in the regulation of the proliferation and migration of PASMCs, and to assess the role of ZIP12 in the PI3K/AKT and ERK signaling cascades in PASMCs, which can enhance our understanding of the pathophysiological role of ZIP12 in PAH.

## Materials and methods

### Antibody production

A ZIP12 monoclonal antibody was generated by HuaAn Biotechnology (Hangzhou, China). A customed antibody against peptides corresponding to the last five amino acids at the C-terminus of both human and rat ZIP12 proteins was produced in rabbits as described by Zhao et al. [15]. Specificity of the rabbit serum containing anti-ZIP12 antibodies was confirmed by Western blot analysis. Only a single band was detected by Western blot at approximately 70 kDa in the lysates of rat lung or PASMCs.

### Animals and PAH model

Male SD rats (200–230 g) were purchased from Shanghai SLACCAS Laboratory Animal Co., Ltd (certificate of quality SCXK 2012-0002). The rats were housed in a common animal room with suitable humidity and temperature. Food and drinking water were available ad libitum. Sixteen rats were randomly assigned to one of two groups: the control group or the PAH group. A PAH model was produced by intraperitoneal injections of 20 mg/kg MCT twice at one-week intervals according to our previously established protocol [18]. Rats in the control group were administered an equal volume of saline intraperitoneally. To minimize animal suffering, all procedures were performed under sodium pentobarbital anesthesia. After 28 days, the rats were sacrificed for hemodynamic measurements and histopathological examination.

### Hemodynamic measurements

Right heart catheterization was performed using a custom-made polyethylene catheter (Peking Union Medical College, Beijing, China) to measure the mean pulmonary arterial pressure (mPAP) as described previously [18]. The rats were euthanized after hemodynamic measurements. The heart and lung tissues were harvested, and histological analysis was performed. The weight of the left ventricle, right ventricle, and septum was measured, and the ratio of right ventricular weight to left ventricular plus septum weight, i.e., Fulton index, was determined.

### Histological analysis

The lung tissues were embedded in a paraffin block, sectioned, and stained with hematoxylin–eosin. Pulmonary arterioles with a diameter less than 200 μm were selected for morphological analysis. Wall thickness (WT), external diameter (ED), luminal area (LA), and total vascular area (TA) were determined using Image-Pro Plus 6.0 software. Remodeling of pulmonary arterioles was evaluated based on the percentage of the vascular wall area (WA%, WA% = (TA − LA)/TA × 100%) and the percentage of vascular wall thickness (WT%, WT% = 2 × WT/ED × 100%). An immunochemistry assay was performed to assess the proportion of muscularization of pulmonary arterioles using an antibody to α-smooth muscle actin (α-SMA) (Abcam, cat. ab7817, 1:100 dilution). The degree of muscularization was defined by the percentage of the length of α-SMA positive staining to the length of the whole vessel circumference (nonmuscularized: < 25% α-SMA staining around the vessel; partially muscularized: 25–75% α-SMA staining around the vessel; and fully muscularized: > 75% α-SMA staining around the vessel). The extent of perivascular fibrosis was determined using Masson staining. Immunohistochemical analysis of proliferating cell nuclear antigen (PCNA) was performed to assess cell proliferation. The nuclei of PCNA-positive cells were stained brown. The numbers of PCNA-positive cells and total cells in the pulmonary arterial media walls were counted. The percentage of positive cell number was calculated as positive cells/total cells. The localization and expression of ZIP12 in the lung tissues were determined by immunofluorescence. ZIP12 fluorescence intensity in pulmonary arteries was estimated as ZIP12 integrated fluorescence intensity divided by the area marked by the α-SMA signal. Further details of these methods have been described previously [19, 20].

### Isolation and culture of PASMCs

Rats were deeply anesthetized with 50 mg/kg sodium pentobarbital and euthanized by cervical dislocation. Primary PASMCs were prepared from pulmonary arteries using a previously described protocol [19, 21]. α-SMA immunofluorescence was used to identify PASMCs. The cells were maintained in Dulbecco’s modified Eagle’s medium/F12 (DMEM/F12, HyClone, USA) supplemented with 10% fetal bovine serum (FBS, Gibco, Australia) and 1% penicillin–streptomycin (HyClone, USA) at 37 °C in an atmosphere of 5% CO_2_. PASMCs isolated from control rats (Ctrl-PASMCs) and MCT-treated rats (MCT-PAH-PASMCs) were used in all experiments.

### Immunofluorescence staining

PASMCs were seeded on sterile coverslips. Prior to immunofluorescence staining, the cells were treated with the cell membrane green fluorescent probe DiO (Solarbio, China) for 10 min. Blocking and antibody incubation were performed without Triton X-100 permeabilization. After blocking with 10% goat serum, the cells were incubated with antibodies against ZIP12 (1:100) overnight. After overnight incubation at 4 °C, the cells were washed three times with 1× PBS, incubated with an Alexa Fluor 594-labeled secondary antibody (Zhongshan Golden Bridge, China, 1:200) for 2 h, and counterstained with DAPI (Cell Signaling Technology, USA, 1 μg/ml). Fluorescence images were obtained using a Zeiss confocal laser scanning microscope (LSM 800, Zeiss, Germany).

### Vector construction and cell infection

A ZIP12 knockdown lentivirus (LV-shZIP12) and an overexpression lentivirus (LV-ZIP12) were designed and constructed by GeneChem (Shanghai, China). For LV-shZIP12, the siRNA sequence of ZIP12, GTC ATG AAA TTC CAC ATG A, was cloned into the hU6-MCS-CBh-gcGFP-IRES-puromycin lentivirus. The negative control scrambled sequence, TTC TCC GAA CGT GTC ACG T, was cloned into the same lentiviral vector (LV-shNC). For LV-ZIP12, the ZIP12 sequence (NM_001106124) was cloned into the Ubi-MCS-3FLAG-CBh-gcGFP-IRES-puromycin lentivirus. The empty lentiviral vector was used as a negative control (LV-NC). For knockdown studies, MCT-PAH-PASMCs were transfected with LV-shZIP12, and for overexpression studies, Ctrl-PASMCs were transfected with LV-ZIP12. The optimal MOI for PASMC infection was determined in a preliminary experiment. After incubation for 72 h, the cells were collected for subsequent studies.

### Western blot

Western blot analysis was performed as described previously [19]. Briefly, protein from PASMCs and lung tissues was extracted using lysis buffer (Beyotime, China) containing 1 mM PMSF (Beyotime, China) and 1% protease inhibitor cocktail (Roche, Switzerland). Equal volumes of protein lysates were separated by SDS-PAGE gels and electroblotted onto PVDF membranes. Then, the PVDF membranes were incubated with primary antibodies at 4 °C overnight after blocking with 5% skimmed milk. Anti-p-ERK1/2 (Cell Signaling Technology, cat. #4370) antibody was diluted 1:10,000; anti-ERK1/2 (Cell Signaling Technology, cat. #4695) and anti-β-actin (Santa Cruz, cat. sc-8432) antibodies were diluted 1:5000, and anti-p-AKT (Cell Signaling Technology, cat. #4060), anti-AKT (Cell Signaling Technology, cat. #4685), and anti-PCNA (Abcam, cat. ab29) antibodies were diluted 1:1000; anti-ZIP12 (HuaAn Biotechnology) and anti-cyclin D1 (Cell Signaling Technology, cat. #55506) antibodies were diluted 1:500. The membranes were extensively washed in TBST and incubated with appropriate secondary antibodies. Finally, protein bands were detected using an ECL kit (Beyotime, China). Relative intensities of the bands were quantified using Image J. The unprocessed images for Western blot were supplied in the Additional file 6.

### Real-time quantitative PCR

Real-time quantitative PCR was performed as the previous description [22, 23]. Briefly, total RNA from the lung tissues was extracted using TRIzol reagent (Invitrogen, USA). Real-time quantitative PCR was carried out using SYBR Green Master Mix (Roche, Switzerland). The primer pairs used for amplification were as follows: ZIP12 F: 5′-GTT ATG GTC CTG GTT GGA GAT G-3′; R: 5′-AAT AGC GAT TGT TGT GGT CAC T-3′; GAPDH F: 5′-ACG GCA AGT TCA ACG GCA CAG-3′; R: 5′-GAA GAC GCC AGT AGA CTC CAC GAC-3′.

### Cell proliferation assay

Cell proliferation was detected using an EdU incorporation assay (Beyotime, China) as described previously [21]. EdU-positive cells (red fluorescence) corresponded to proliferating cells, and Hoechst 33342-stained cells (blue fluorescence) corresponded to all cells. The cell proliferation rate was calculated as the ratio of EdU-positive cells to all cells.

### Wound healing assay

The migration of PASMCs after various treatments was assessed by wound healing assay. PASMCs in each group were cultured to 90% confluence in 6-well plates. A straight line was produced by scratching using a 200 μl pipette tip at the bottom of each well. Images of the scratches were captured at 0 h, 24 h, and 48 h. The cell migration rate was quantified using the equation: (area of initial scratch − area of current scratch)/area of initial scratch × 100%.

### Statistical analysis

Data are presented as the mean ± standard deviation and were analyzed with GraphPad Prism 7. Comparisons between two groups were performed by using Students t-test, and three or more groups were compared using one-way ANOVA, followed by Dunnett test or Tukey test, as appropriate. All tests were two-sided, and *P* < 0.05 indicated statistical significance.

## Results

### Hemodynamic measurements and morphometric analysis of pulmonary arterioles

Hemodynamics were examined via right heart catheterization 28 days after administration of the first dose of MCT. Representative pulmonary artery waveforms are presented in Additional file 1: Fig. S1A. MCT-treated rats manifested a significant increase in mPAP compared with that in control rats (Additional file 1: Fig. S1A). Furthermore, a significant increase in Fulton index was observed 4 weeks after MCT exposure (Additional file 1: Fig. S1B). The results of HE staining of the lung tissue indicated that thickness of the arterial wall in the control group was low and endothelial cells were intact, without necrosis and inflammatory cell infiltration. Comparison with the control group indicated that the lung structure was disordered in the MCT-PAH group, with thickening of the intima accompanied by massive inflammatory cell infiltration (Additional file 1: Fig. S1C). Furthermore, WA% and WT% of pulmonary arterioles in the MCT-PAH group were significantly higher than those in the control group (Additional file 1: Fig. S1C). To assess the degree of pulmonary vascular remodeling, immunohistochemistry of α-SMA was performed to evaluate the degree of muscularization of pulmonary arterioles. As shown in Additional file 1: Fig. S1D, a significant increase in muscularization of distal pulmonary arterioles in the MCT-PAH group was observed compared with that in the control group. Pulmonary arterial remodeling in the MCT-induced PAH rat model was associated with extensive proliferation of pulmonary arterial smooth muscle cells. To assess cell proliferation, we performed immunohistochemical staining for PCNA, a marker of cellular proliferation, in rat lung tissue samples. As shown in Additional file 1: Fig. S1E, rats with MCT-induced PAH manifested an increase in the number of PCNA-positive cells in the vascular smooth muscle of pulmonary arterioles. Collagen deposition is another important feature of pulmonary vascular remodeling. MCT treatment increased collagen deposition, as shown by blue positive area of Masson staining of pulmonary vessel walls (Additional file 1: Fig. S1F).

### The expression of ZIP12 and MCT-induced proliferation in vivo

**Fig. 1:**
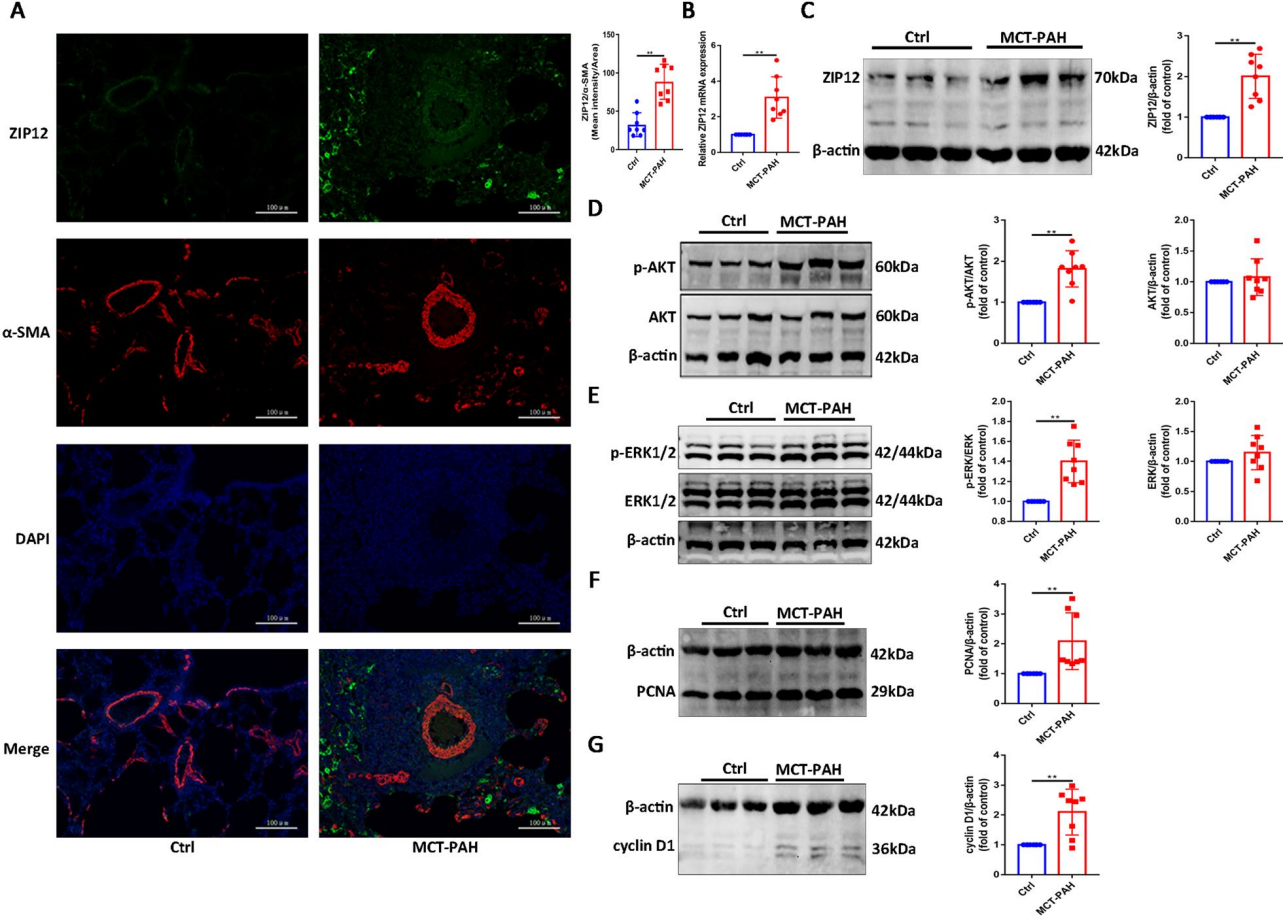
The expression of ZIP12 and MCT-induced proliferation in vivo. Immunofluorescence analysis were performed. **A** Representative images of double immunofluorescence staining for ZIP12 (green) and α-SMA (red) in lung tissues (magnification, ×200) and quantification of ZIP12 immunofluorescence intensity (n = 8 rats per group). **B** ZIP12 mRNA expression in lung tissues (n = 8 rats per group). Representative Western blot and summarized data showing the protein expression of **C** ZIP12, **D** phosphorylated and total AKT, **E** phosphorylated and total ERK1/2, **F** PCNA and **G** cyclin D1 in rat lung tissues (n = 8 rats per group). *Ctrl* control, *PAH* pulmonary arterial hypertension, *MCT* monocrotaline. The data are expressed as the mean ± standard deviation. Error bars represented standard deviation. **P < 0.01

To determine whether the expression of ZIP12 is associated with MCT‐induced pulmonary vascular remodeling, lung samples from rats with MCT-induced PAH were collected and stained for ZIP12 by immunofluorescence. The results of the dual immunofluorescence staining revealed that ZIP12 expression was increased and partially colocalized with α-SMA in the lung tissue sections of rats with MCT-induced PAH (Fig. 1A). Then, total RNA was extracted from the lung tissues, and the expression of ZIP12 mRNA was measured by RT-qPCR. A significant elevation in ZIP12 mRNA in the lung tissues of rats with MCT-induced PAH compared with that in normal controls was shown (Fig. 1B). The data of Western blot analysis showed that the protein level of ZIP12 was consistent with the mRNA level (Fig. 1C).

Moreover, it was shown that the development of PAH was associated with a significant increase in the levels of p-AKT and p-ERK1/2 proteins in the lung tissues, without changes in the levels of total AKT and ERK1/2 (Fig. 1D, E). Because PCNA and cyclin D1 play a major role in the cell cycle, expression of these proteins in the lung tissues was also analyzed. A significant increase in the protein levels of PCNA and cyclin D1 was observed in the MCT-PAH group compared with those in the control group (Fig. 1F, G).

### MCT treatment enhanced the expression of ZIP12 in PASMCs

**Fig. 2:**
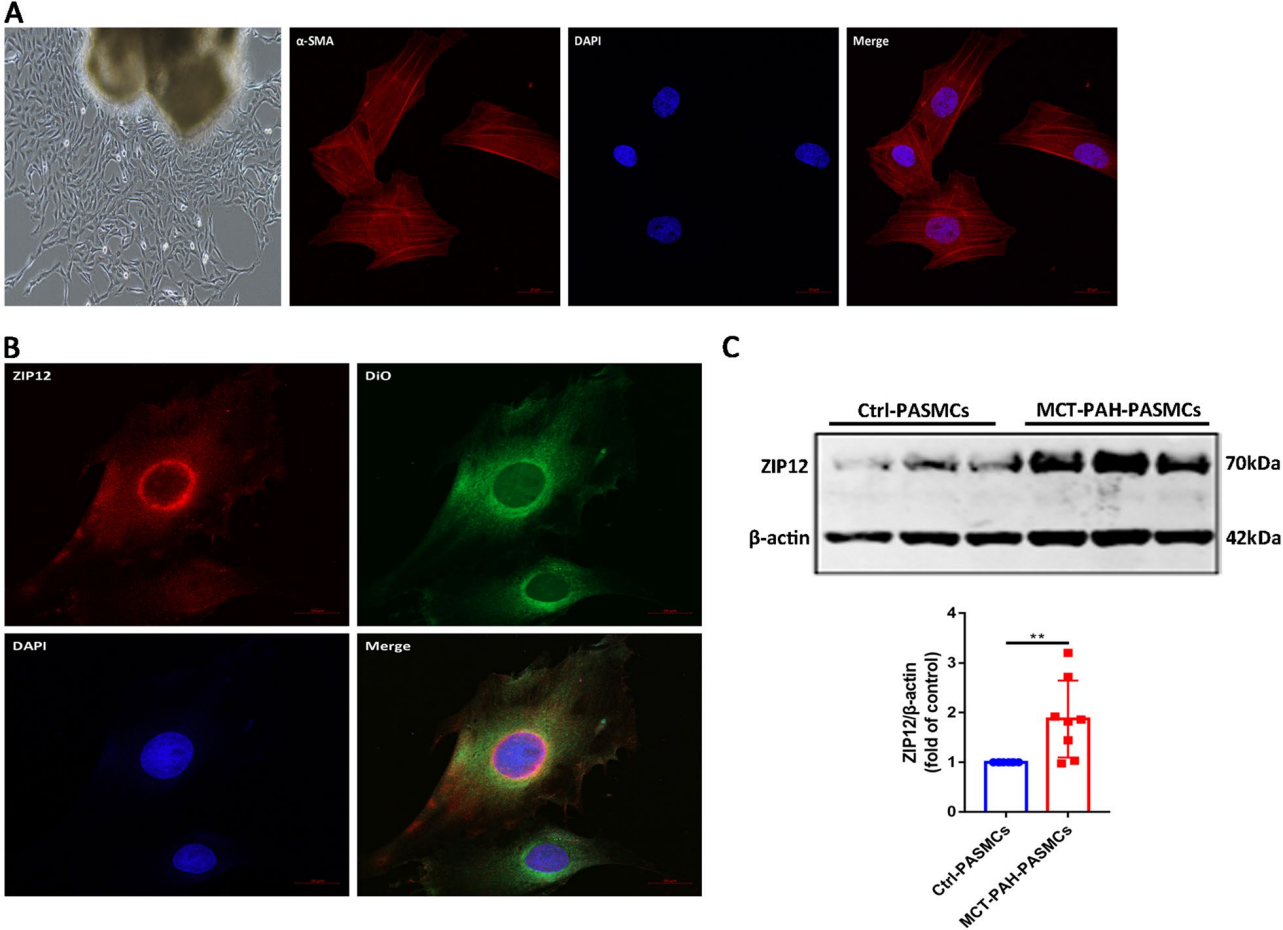
MCT treatment enhanced the expression of ZIP12 in PASMCs. **A** Rat PASMCs exhibited long spindle-shape (magnification, ×100) and were identified by immunofluorescence staining for α-SMA (magnification, ×400). **B** ZIP12 (red) colocalized with the cell membrane dye DiO (green) in PASMCs (magnification, ×200). **C** Representative Western blot and summarized data showing the protein expression of ZIP12 in Ctrl-PASMCs and MCT-PAH-PASMCs (n = 8 rats per group). *Ctrl* control, *PAH* pulmonary arterial hypertension, *PASMCs* pulmonary arterial smooth muscle cells, *MCT* monocrotaline. The data are expressed as the mean ± standard deviation. Error bars represented standard deviation. **P < 0.01

Primary PASMCs appeared spindle-shaped, and their purity (> 98%) was determined by immunofluorescence staining with a specific antibody against α-SMA (Fig. 2A). DiO is a green-fluorescent lipophilic dye that can be used to stain the cell membranes. PASMCs were stained with an anti-ZIP12 antibody followed by a secondary antibody labeled with Alexa Fluor 594. Colocalization of ZIP12 with DiO in the cell membranes resulted in an overlap of the red and green signals corresponding to yellow fluorescence in the cell membranes (Fig. 2B). To investigate whether ZIP12 is involved in the pathogenesis of MCT-induced PAH, the protein expression of ZIP12 in PASMCs was detected. PASMCs from control (Ctrl-PASMCs) or MCT-treated rats (MCT-PAH-PASMCs) were cultured. There was a significant elevation of ZIP12 expression in MCT-PAH-PASMCs compared with that in Ctrl-PASMCs (Fig. 2C).

### MCT treatment increased the proliferation and migration of PASMCs

Excessive proliferation and migration of PASMCs is an essential step of pathogenic pulmonary vascular remodeling in PAH; hence, the biological behavior of Ctrl-PASMCs and MCT-PAH-PASMCs was compared. The EdU assay indicated that MCT-PAH-PASMCs had a higher rate of proliferation compared with that of Ctrl-PASMCs (Additional file 2: Fig. S2A). In addition to proliferation, the increased migration of MCT-PAH-PASMCs was noted based on the results of the wound healing assays (Additional file 2: Fig. S2B). Upregulation of PCNA and cyclin D1 was found suggesting excessive proliferation of MCT-PAH-PASMCs (Additional file 2: Fig. S2C–D).

### The phosphorylation of AKT and ERK1/2 were enhanced in MCT-PAH-PASMCs upon stimulation with 10% FBS

The levels of p-AKT, AKT, p-ERK1/2, and ERK1/2 in Ctrl-PASMCs and MCT-PAH-PASMCs were compared by Western blot analysis at various time points after stimulation with 10% FBS for 0 min, 5 min, 15 min, 30 min, and 60 min. As shown in Additional file 3: Fig. S3, the levels of p-AKT/AKT and p-ERK/ERK reached peaks at 5 min and 15 min, respectively, and gradually decreased up to 60 min after stimulation of Ctrl-PASMCs with 10% FBS. However, an increase in the p-AKT/AKT and p-ERK/ERK levels reached a maximum within 5 min and 15 min, respectively, and persisted at this maximal level for 60 min after stimulation with 10% FBS in MCT-PAH-PASMCs. Total levels of AKT and ERK1/2 did not change significantly between the two groups at various time points. These results indicated that the activity of AKT and ERK1/2 was substantially enhanced in response to stimulation with 10% FBS in MCT-PAH-PASMCs compared with that in Ctrl-PASMCs.

### Transfection efficiency and the expression of ZIP12 after the transfection

ZIP12 was upregulated in MCT-PAH-PASMCs; hence, we used a lentivirus encoding shRNA targeting ZIP12 (LV-shZIP12) to silence the expression of ZIP12 in MCT-PAH-PASMCs for functional studies. To determine optimal MOI for the infection, MCT-PAH-PASMCs were transfected with LV-shZIP12 at various MOI (MOI = 0, 1, 5, 10, 50, and 100) and cultured for 72 h. The results indicated that transduction efficiency of the lentivirus was more than 95% at MOI = 50 (Additional file 4: Fig. S4A–B). Most cells appeared to die at MOI = 100. A significant reduction in ZIP12 protein expression was shown in Western blot analysis when MOI was 50 in MCT-PAH-PASMCs (Additional file 4: Fig. S4C). Consequently, MOI of 50 was used in subsequent experiments.

Similarly, Ctrl-PASMCs were transfected with ZIP12 overexpression lentivirus (LV-ZIP12) at various MOI (MOI = 0, 1, 5, 10, 50, and 100) and cultured for 72 h. The results of flow cytometry analysis suggested that at MOI 50, the infection efficiency was over 95% (Additional file 4: Fig. S4A–B). Notably, when MOI was 100, the cells lost their normal morphological features, and extracellular spaces were widened. The results were confirmed by densitometric quantification of Western blot, suggesting that cells transfected with LV-ZIP12 manifested significantly higher expression of ZIP12 at the protein level (Additional file 4: Fig. S4D). Therefore, MOI 50 was selected for overexpression experiments.

### ZIP12 knockdown decreased the proliferation and migration of MCT-PAH-PASMCs

**Fig. 3:**
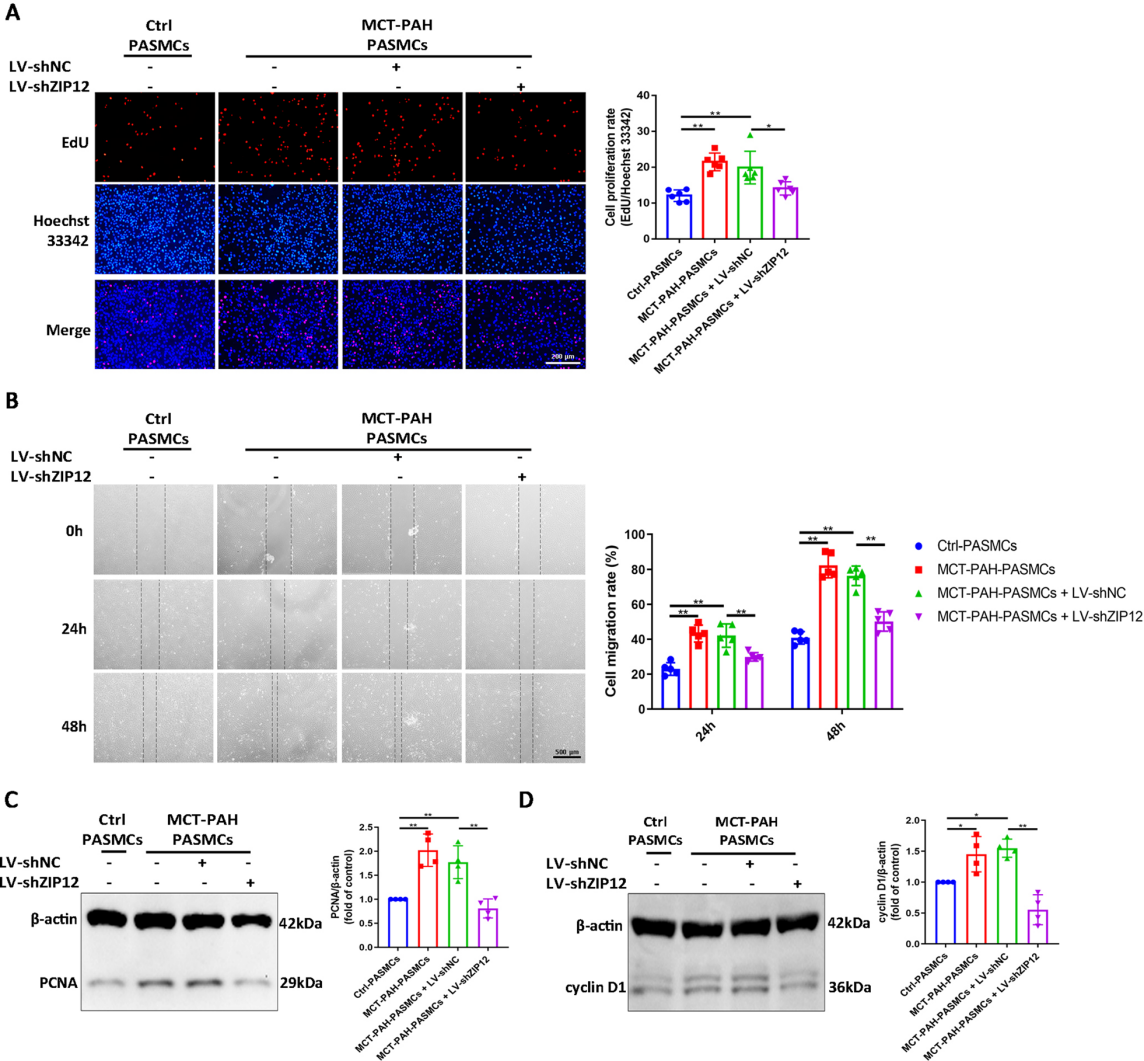
ZIP12 knockdown decreased the proliferation and migration of MCT-PAH-PASMCs. MCT-PAH-PASMCs were infected with LV-shNC or LV-shZIP12. After 72 h of infection, the cells were serum starved for 24 h in 0.2% FBS in DMEM-F12, followed by restimulation with 10% FBS in DMEM-F12 for 48 h. **A** The proliferation of MCT-PAH-PASMCs in response to ZIP12 knockdown was determined by EdU assay (magnification, ×100; n = 6 independent experiments). **B** The migration of MCT-PAH-PASMCs in response to ZIP12 knockdown was determined by wound healing assay. The wounds were imaged every 24 h (magnification, ×40; n = 5 independent experiments). **C**, **D** Representative Western blot and summarized data of ZIP12 silencing and the effect on **C** PCNA and **D** cyclin D1 protein expression (n = 4 independent experiments). *Ctrl* control, *PAH* pulmonary arterial hypertension, *PASMCs* pulmonary arterial smooth muscle cells, *EdU* 5-ethynyl-2′deoxyuridine, *MCT* monocrotaline. The data are expressed as the mean ± standard deviation. Error bars represented standard deviation. *P < 0.05, **P < 0.01

To further explore the biological role of ZIP12, the effect of ZIP12 downregulation on the proliferation and migration of MCT-PAH-PASMCs was investigated. MCT-PAH-PASMCs were transfected either with LV-shNC or LV-shZIP12, serum-starved for 24 h, and stimulated with 10% FBS for 48 h. EdU assays demonstrated that the rates of proliferation of MCT-PAH-PASMCs were increased compared with that of Ctrl-PASMCs and were inhibited by silencing ZIP12 (Fig. 3A). Moreover, the data of the scratch assays indicated that ZIP12 silencing inhibited the migration of MCT-PAH-PASMCs (Fig. 3B). Consistently, an increase in the expression of PCNA and cyclin D1 in MCT-PAH-PASMCs was dramatically attenuated after ZIP12 silencing (Fig. 3C, D).

### ZIP12 overexpression promoted the proliferation and migration of Ctrl-PASMCs

To define the function of ZIP12 in the regulation of cell proliferation and migration, ZIP12 overexpression lentivirus was generated and transfected into Ctrl-PASMCs. Comparatively, overexpression of ZIP12 increased the proliferation (Additional file 5: Fig. S5A) and migration (Additional file 5: Fig. S5B) and expression of PCNA and cyclin D1 (Additional file 5: Fig. S5C–D) in Ctrl-PASMCs stimulated with 10% FBS. These results confirmed the function of ZIP12 as a positive regulator of the proliferation and migration in PASMCs.

### Silencing ZIP12 suppressed the phosphorylation of AKT and ERK1/2 in MCT-PAH-PASMCs

**Fig. 4:**
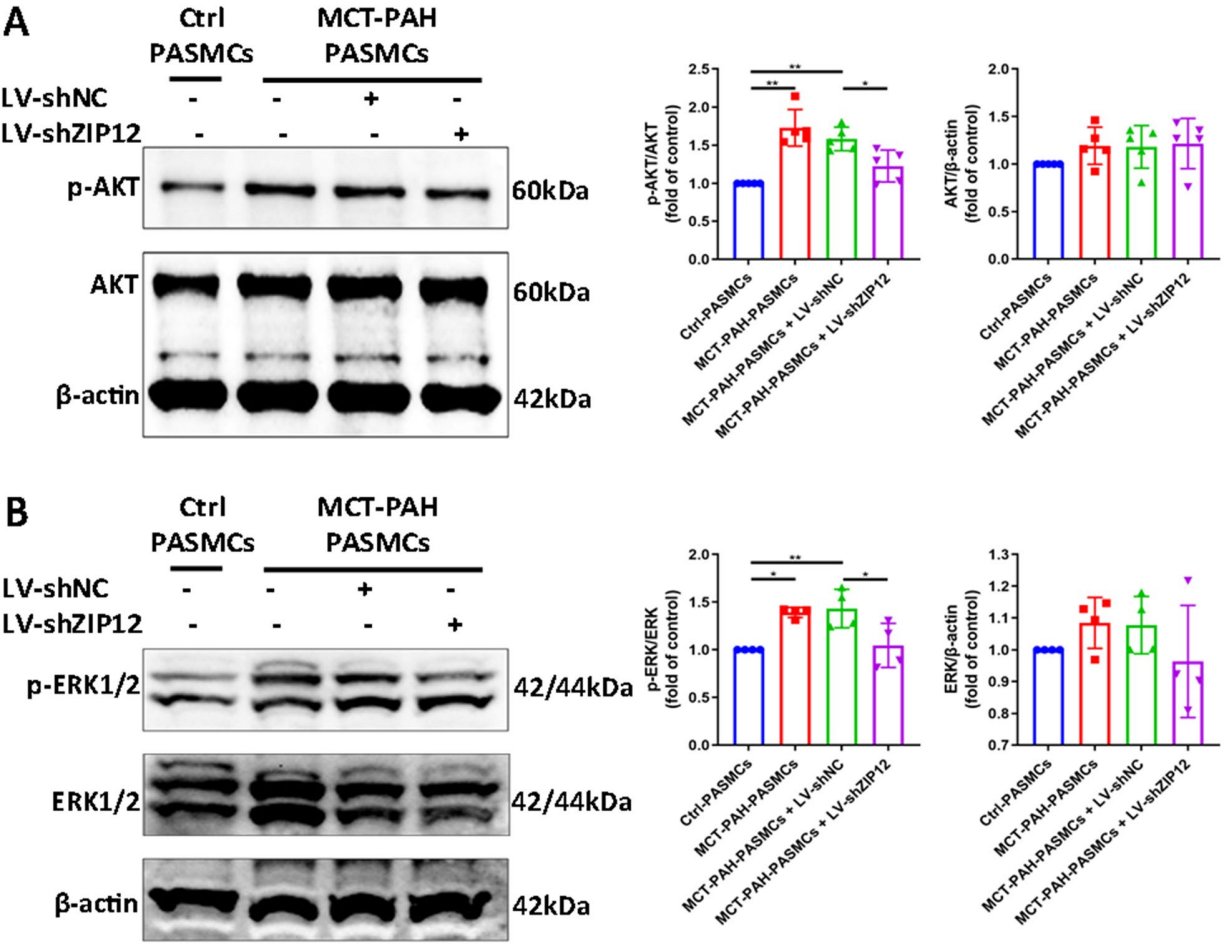
Silencing ZIP12 suppressed the phosphorylation of AKT and ERK1/2 in MCT-PAH-PASMCs. MCT-PAH-PASMCs were infected with LV-shNC or LV-shZIP12. After 72 h of infection, the cells were serum starved for 24 h in 0.2% FBS in DMEM-F12, followed by restimulation with 10% FBS in DMEM-F12 for 1 h. **A**, **B** Representative Western blot and summarized data showing the protein expression of **A** phosphorylated and total AKT and **B** phosphorylated and total ERK1/2 in MCT-PAH-PASMCs after ZIP12 silencing (**A**, n = 5 independent experiments; **B**, n = 4 independent experiments). *Ctrl* control, *PAH* pulmonary arterial hypertension, *PASMCs* pulmonary arterial smooth muscle cells, *MCT* monocrotaline. The data are expressed as the mean ± standard deviation. *P < 0.05, **P < 0.01

To provide insight into the molecular mechanisms responsible for ZIP12-mediated proliferation and migration of PASMCs, we focused on the PI3K/AKT and ERK signaling pathways, which are critical for cellular proliferation and migration. The role of ZIP12 in the activation of the PI3K/AKT and ERK signaling pathways in PASMCs was investigated by silencing ZIP12 gene. As illustrated in Fig. 4, ZIP12 gene silencing inhibited phosphorylation of AKT and ERK1/2 induced by 10% FBS in MCT-PAH-PASMCs without altering total levels of AKT and ERK1/2.

### Overexpression of ZIP12 promoted the phosphorylation of AKT and ERK1/2 in Ctrl-PASMCs

**Fig. 5:**
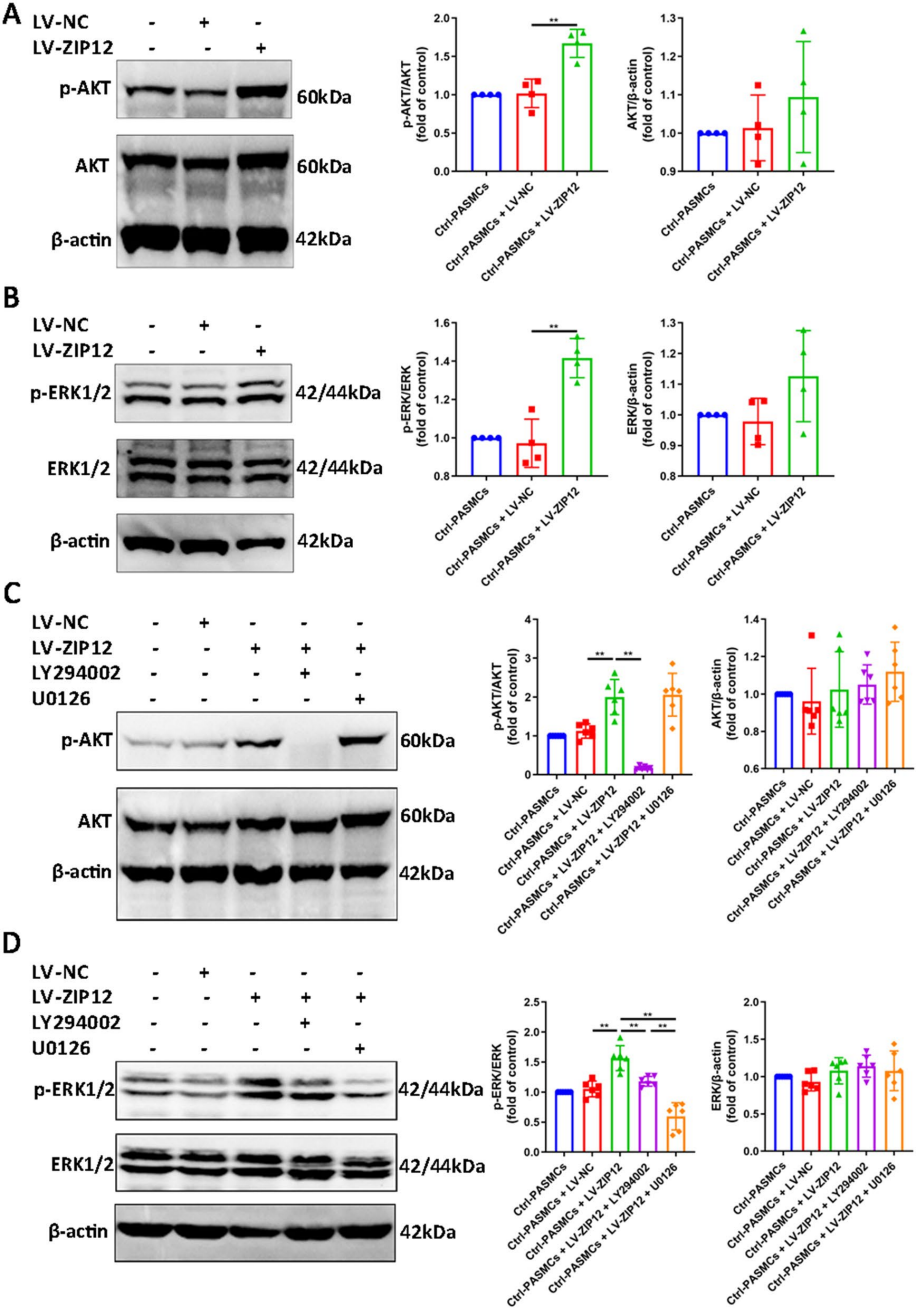
Overexpression of ZIP12 promoted the phosphorylation of AKT and ERK1/2 in Ctrl-PASMCs. **A**, **B** Ctrl-PASMCs were infected with LV-NC or LV-ZIP12. After 72 h of infection, cells were serum starved for 24 h in 0.2% FBS/DMEM-F12, followed by restimulation with 10% FBS/DMEM-F12 for 1 h. Representative Western blot and summarized data showing the protein expression of **A** phosphorylated and total AKT and **B** phosphorylated and total ERK1/2 in Ctrl-PASMCs after ZIP12 overexpressing (**A**, n = 4 independent experiments; **B**, n = 4 independent experiments). **C**, **D** Ctrl-PASMCs were infected with LV-NC or LV-ZIP12. After 72 h of infection, cells were serum starved for 24 h in 0.2% FBS/DMEM-F12, and then pretreated with 10 μM LY294002 or 10 μM U0126 for 30 min prior to restimulation with 10% FBS/DMEM-F12 for 1 h. Representative Western blot and summarized data showing the protein expression of **C** phosphorylated and total AKT and **D** phosphorylated and total ERK1/2 in ZIP12-overexpressing Ctrl-PASMCs treated with either ERK inhibitor U0126 or AKT inhibitor LY294002, or untreated (**C**, n = 6 independent experiments; **D**, n = 6 independent experiments). *Ctrl* control, *PASMCs* pulmonary arterial smooth muscle cells. The data are expressed as the mean ± standard deviation. Error bars represented standard deviation. **P < 0.01

Then, we investigated whether in vitro overexpression of ZIP12 enhances the activation of the PI3K/AKT and ERK signaling pathways. As shown in Fig. 5A, B, ZIP12 overexpression enhanced both AKT and ERK1/2 phosphorylation in Ctrl-PASMCs stimulated with 10% FBS. To determine potential relationships between AKT and ERK1/2, ZIP12-overexpressing Ctrl-PASMCs were pretreated with the AKT inhibitor LY294002 or ERK1/2 inhibitor U0126. The results of Western blot indicated that U0126 administration significantly attenuated ERK1/2 activation without affecting AKT phosphorylation (Fig. 5C, D). Interestingly, LY294002 treatment completely abolished AKT activation, and the phosphorylation of ERK1/2 was also partially inhibited (Fig. 5C, D).

### ZIP12 promoted the proliferation and migration of PASMCs via the AKT/ERK signaling pathways

**Fig. 6:**
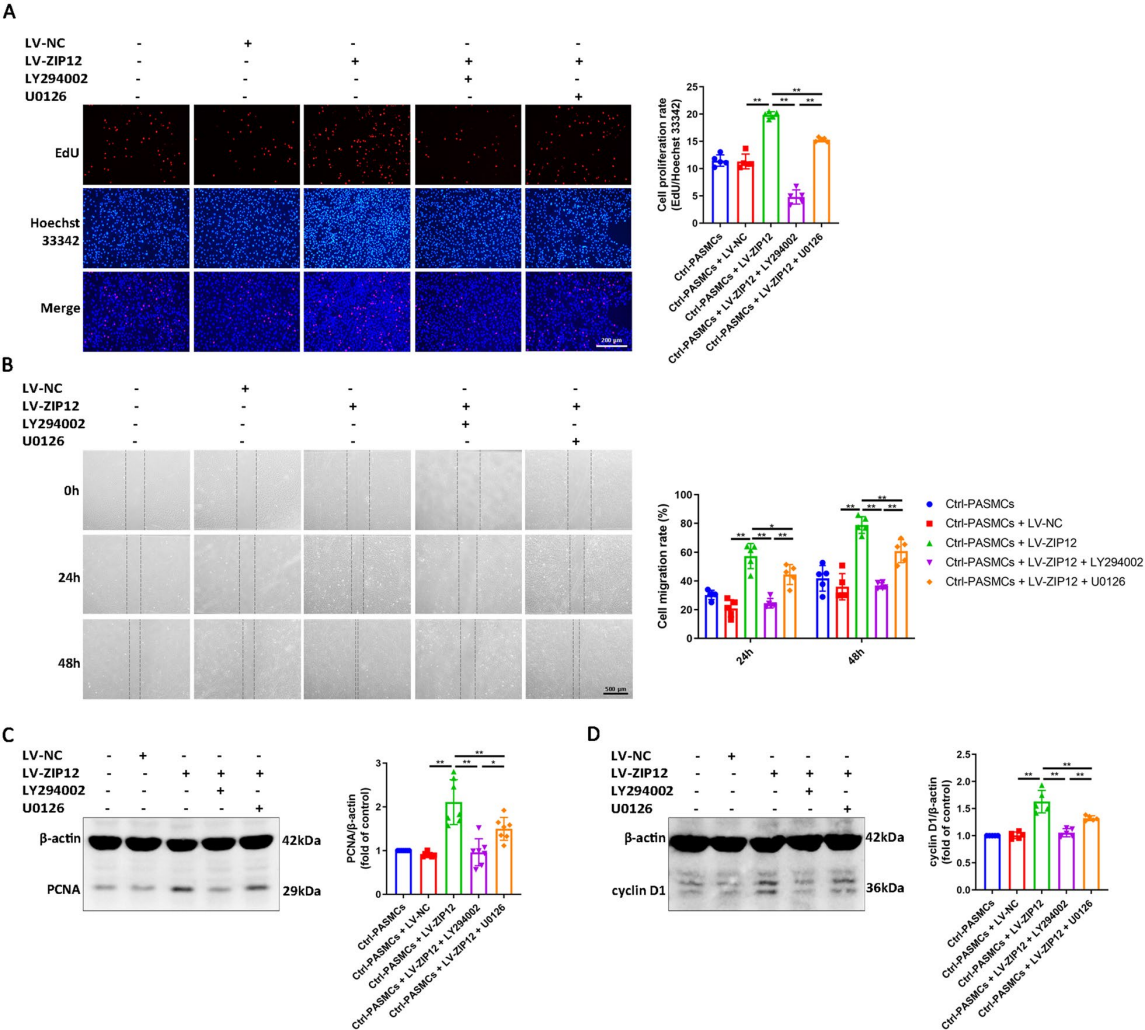
ZIP12 promoted the proliferation and migration of PASMCs via the AKT/ERK signaling pathways. Ctrl-PASMCs were infected with LV-NC or LV-ZIP12. After 72 h of infection, cells were serum starved for 24 h in 0.2% FBS/DMEM-F12, and then pretreated with 10 μM LY294002 or 10 μM U0126 for 30 min prior to restimulation with 10% FBS/DMEM-F12 for 48 h. **A** Cell proliferation was determined by EdU assay (magnification, ×100; n = 5 independent experiments). **B** Cell migration was determined by wound healing assay. The wounds were imaged every 24 h (magnification, ×40; n = 5 independent experiments). **C**, **D** Representative Western blot and semiquantitative analysis of **C** PCNA and **D** cyclin D1 protein in each group (**C**, n = 7 independent experiments; **D**, n = 5 independent experiments). *Ctrl* control, *PASMCs* pulmonary arterial smooth muscle cells, *EdU* 5-ethynyl-2′deoxyuridine. The data are expressed as the mean ± standard deviation. Error bars represented standard deviation. *P < 0.05, **P < 0.01

To investigate the exact contribution of the AKT and ERK activation to enhanced cell proliferation and migration triggered by ZIP12 overexpression, we used LY294002 and U0126 to block the activation of AKT and ERK, respectively. The results demonstrated that an increase in the proliferation and migration of ZIP12-overexpressing PASMCs disappeared completely or partially when the cells were pretreated with LY294002 or U0126, respectively (Fig. 6A, B). An increase in the expression of PCNA and cyclin D1 due to ZIP12 overexpression was significantly inhibited by pretreatment with LY294002 or U0126 (Fig. 6C, D).

## Discussion

The present study investigated the expression pattern, biological function, and molecular mechanism of action of ZIP12 in MCT-induced PAH. It was found that the level of ZIP12 expression was significantly elevated in the lung tissues and isolated PASMCs from the MCT-induced PAH rats in this study. In vitro experiments demonstrated that overexpression of ZIP12 facilitated the proliferation and migration and enhanced the phosphorylation of AKT and ERK1/2 in Ctrl-PASMCs, whereas silencing ZIP12 expression had the opposite effects in MCT-PAH-PASMCs. Moreover, inhibition of the PI3K/AKT signaling pathway abolished the effect of ZIP12 overexpression on enhancing cell proliferation and migration and partially suppressed an increase in ERK1/2 phosphorylation, which was induced by overexpression of ZIP12. However, cell proliferation and migration induced by ZIP12 overexpression was only partially reversed by blockade of the ERK signaling pathway, whereas the phosphorylation of AKT was basically unaffected. This study provides novel insight into the regulatory mechanism of ZIP12 in the proliferation and migration of PASMCs and reveals a new potential therapeutic strategy for PAH.

MCT is a pyrrolizidine alkaloid isolated from the seeds or leaves of Crotalaria spectabilis plant [24]. A rat model of MCT-induced PAH has emerged as the most frequently used animal model of PAH. Comparison with other PAH models, the main advantage of MCT-induced PAH model involves an imitation of several key features of human PAH, including impairment of endothelial function, proliferation of smooth muscle cells, and high inflammatory status [25]. Pulmonary vascular wall remodeling resulting from unrestricted and excessive proliferation and migration of PASMCs is the main pathological feature of PAH. PCNA and cyclin D1 have been identified as molecular markers of cell proliferation. PCNA acts as a sliding clamp for replicative DNA polymerases and was shown to participate in DNA synthesis and DNA damage repair [26]. Based on the above main pathogenesis of MCT-induced PAH, this study was carried out to explore the role of ZIP12 in the developments and progression during the PAH and underlying mechanism. Our present study indicated that the level of PCNA protein was significantly increased in the lung tissues of rats with MCT-induced PAH. An increase in cellular proliferation was observed in smooth muscle cells, as determined by PCNA immunohistochemistry. Cyclin D1 is an important subunit of cyclin-dependent kinases (CDKs) [27]. Cyclin D1 forms a complex with CDK4 or CDK6, inactivating the Rb protein and leading to the promotion of the G1/S phase transition [28]. shRNA targeting cyclin D1 was reported to attenuate MCT‐induced pulmonary vascular remodeling [29]. Our results also indicated an increase in cyclin D1 expression in the lung tissues from rats with MCT-induced PAH. Moreover, PASMCs isolated from MCT-treated rats manifested increased proliferation and migration compared with that of PASMCs isolated from control rats. These results showed that PASMCs undergo significant alterations in their functional properties during the pathogenesis of MCT-induced PAH, which is, to a certain extent, similar to cancer cells.

Potential importance of zinc transport proteins in pulmonary vascular disease has recently emerged only in the field of basic research. ZIP12 is a membrane-spanning protein that transports zinc ions into the cytoplasm from the extracellular space. The present study confirmed the localization of ZIP12 in the cell membranes. RAC1 is a member of the Rho small G protein family involved in the formation of membrane ruffles [30]. Colocalization of ZIP12 and RAC1 in PASMCs has been reported previously [31]. A recent study reported an elevated expression levels of ZIP12 in many cell types, including pulmonary interstitial, smooth muscle, and endothelial cells, in distal pulmonary arterioles of rats, calves, and humans susceptible to pulmonary hypertension due to chronic hypoxia [15]. Knockdown of ZIP12 inhibits hypoxia-induced influx of zinc ions into PASMCs and subsequent proliferation of the cells, ameliorates pulmonary arterial remodeling, and suppresses elevated pulmonary arterial pressure [15]. Moreover, it has recently been shown that ZIP12 plays an important role in the hypoxia-induced phenotypic transition of PASMCs [32]. In our recently published work, we demonstrated that elevated intracellular labile zinc possibly from ZIP12 was associated with increased CREB-mediated transcriptional activity and PASMCs proliferation [16]. However, functional characterization is further required for ZIP12 via the transgenic approaches including the gene overexpression and silencing analysis. Consistent with previous studies [15–17], our results indicated a considerable increase in ZIP12 expression in pulmonary arterioles of rats with MCT-induced PAH. Additionally, our study indicated that the expression level of ZIP12 was considerably elevated in MCT-PAH-PASMCs. These preliminary findings suggested that high expression of ZIP12 may be associated with high proliferation and migration of PASMCs and pulmonary vascular remodeling.

Increasing evidence indicated that zinc transport proteins are essential for carcinogenesis and tumor progression, including enhanced proliferation and invasion, in a variety of cancers [33–38]. Zinc is an essential trace element for structural stabilization and activation of a large number of enzymes and transcription factors, and homeostasis of this essential element is strictly regulated by zinc transport proteins [7]. Previous studies showed that zinc plays an important role in cell growth and proliferation possibly by influencing DNA replication, protein synthesis and the cell cycle [39]. To date, few information about a possible role of ZIP12 in mammalian cell proliferation and migration is reported. The present study explored the function of ZIP12 in PASMCs by performing gain-of-function and loss-of-function experiments. Suppression of ZIP12 reduced the proliferation and migration of MCT-PAH-PASMCs, and overexpression of ZIP12 promoted the proliferation and migration of Ctrl-PASMCs. Notably, this is the first report describing the effect of ZIP12 on the migration of PASMCs. The results of the present study indicated that ZIP12 functions as a positive regulator of the proliferation and migration of PASMCs.

To some extent, the biological patterns involved in PAH share several features with cancer, including unrestrained cell proliferation, enhanced migration, and resistance to apoptosis [40]. High proliferation and migration of PASMCs have been associated with the PI3K/AKT and ERK signaling pathways [41–45]. PI3K is a cytoplasmic lipid kinase involved in the regulation of a variety of processes that are critical for cell survival. AKT is considered as the major downstream effector of PI3K. Phosphorylation at Ser473 allosterically activates AKT and initiates the downstream regulatory activity [46]. MAPK cascades are critical for the proliferation and migration of multiple cell types. ERK1/2 is the first characterized member of the MAPK family and has been extensively studied [47]. The present study demonstrated that the levels of phosphorylated AKT and ERK1/2 in the lung tissues of rats with MCT-induced PAH were significantly increased. Additionally, we compared the phosphorylation status in Ctrl-PASMCs and MCT-PAH-PASMCs in response to stimulation with 10% FBS. In the absence of 10% FBS, almost complete dephosphorylation of AKT and ERK1/2 was observed in both Ctrl-PASMCs and MCT-PAH-PASMCs, which is consistent with previous findings [48, 49]. AKT and ERK1/2 are the major mediators downstream of growth factor receptor signaling [50]. Thus, in the starvation state, AKT and ERK1/2 tend to be inactive. Under 10% FBS stimulation, strong and long-lasting activation of AKT and ERK1/2 was observed in MCT-PAH-PASMCs compared with that in Ctrl-PASMCs. Protein phosphorylation is an important posttranslational modification and is under the control of protein kinases and protein phosphatases [51]. These results may be due in part to a change in the balance between kinase and phosphatase activities. These data imply that the PI3K/AKT and ERK signaling pathways were overactivated in rats with PAH and may be involved in PAH-related vascular remodeling.

To gain deep insight into the potential mechanisms and downstream intracellular signaling cascade by which ZIP12 promotes the proliferation and migration of PASMCs, we investigated the PI3K/AKT and ERK signaling pathways, which are known to promote the proliferation and migration of the cells. The results indicated that overexpression of ZIP12 enhanced the phosphorylation of AKT and ERK1/2 induced by 10% FBS and resulted in increased proliferation and migration of Ctrl-PASMCs. Interestingly, the phosphorylation of AKT was not altered after treatment with the ERK1/2 inhibitor U0126, indicating that ERK1/2 is positioned downstream of AKT. Previous studies have shown that AKT activates ERK1/2 by phosphorylating Raf, and a feedback loop may exist between AKT and ERK1/2 [52]. Crosstalk between AKT and ERK1/2 involving the intracellular signaling in response to the zinc fluctuations should be further studied. Functional rescue experiments also demonstrated that treatment with LY294002 or U0126 completely or partially offset the effect mediated by ZIP12 overexpression on the proliferation and migration of PASMCs. Additionally, silencing ZIP12 in MCT-PAH-PASMCs inhibited AKT and ERK1/2 phosphorylation induced by 10% FBS, further confirming the stimulatory effect on the PI3K/AKT and ERK signaling pathways. An increase in plasma membrane localization of ZIP6 has been reported to enhance AKT phosphorylation by increasing zinc influx [38]. Additionally, activation of ZIP7 induces the release of zinc from intracellular stores and prolongs the activation of several downstream signaling pathways, including MAPK, PI3K, and mTOR [53]. Most recently, Zhu et al. [32] reported that ZIP12 may promote phenotypic switching of PASMCs by activating the ERK signaling pathway under hypoxic conditions. However, in their study, no rescue experiments were performed to verify that these effects of ZIP12 on PASMCs were indeed mediated by ERK1/2. Zinc functions as a secondary messenger involved in cell signaling, and the cytoplasmic levels of zinc are strictly controlled by zinc transport proteins [7]. Zinc can induce the phosphorylation of numerous receptor tyrosine kinases, including insulin receptor (IR), insulin‐like growth factor‐1 receptor (IGF-1R), and epidermal growth factor receptor (EGFR), leading to the activation of the PI3K/AKT and ERK signaling pathways [54, 55]. Moreover, zinc inhibits the activities of multiple protein phosphatases, including protein tyrosine phosphatases (PTPs) [56, 57], protein phosphatase 2A (PP2A) [58, 59], phosphatase and tensin homolog (PTEN) [8, 9], and dual‐specificity phosphatases (DUSPs) [10]. Zinc is able to stimulate the PI3K/AKT pathway by inhibiting the activity of PTEN [8, 9]. Similarly, zinc can promote the activation of the ERK pathway through inhibition of DUSP activity [10]. This effect indicates the presence of a potential synergy between zinc signaling and protein phosphorylation. Moreover, the expression of PCNA and cyclin D1 was also examined in response to ZIP12 overexpression or knockdown. The results indicated that ZIP12 knockdown suppressed the expression of PCNA and cyclin D1 in MCT-PAH-PASMCs. In contrast, ZIP12 overexpression had an opposite effect, and this effect was decreased by LY294002 or U0126 treatment. The expression of cyclin D1 is regulated by a variety of upstream signals, including PI3K/AKT [60], ERK [61], and PKC [62]. Activated AKT is able to maintain the stability of cyclin D1 by suppressing the activity of GSK-3β, a suppressor of cyclin D1 [60]. Furthermore, the activation of the PI3K/AKT and ERK signaling pathways promotes the expression of cyclin D1 by facilitating the binding of activator protein-1 (AP-1) to the promoter of cyclin D1 to induce transcription [61]. And this intracellular signaling pathway may also be the case in the response of intracellular zinc influx in the process of PAH.

Notably, the present study has some limitations. First, the lack of intracellular free zinc quantification assay is the major limitation of the study. Experiments toward these aims are currently in progress, and the results will be reported in future studies. Second, regulation of other signaling pathways by ZIP12 requires further research. Third, implication of other zinc transporter proteins in the regulation of proliferation and migration of PASMCs requires additional studies. Finally, the present study did not explore the therapeutic potential of ZIP12 knockdown in vivo. Future studies using rats with pulmonary vascular smooth muscle‐specific knockout of ZIP12 will be useful for testing the value of ZIP12 as a therapeutic target in PAH.

## Conclusions

**Fig. 7:**
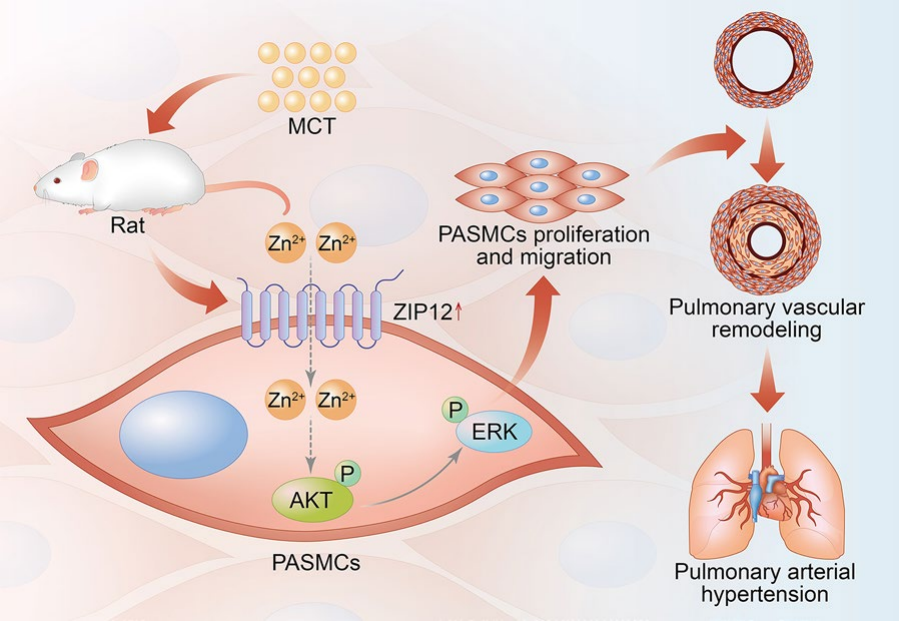
Schematic illustration of the potential role of ZIP12 in the pathogenesis of MCT-induced PAH. *MCT* monocrotaline, *PAH* pulmonary arterial hypertension, *PASMCs* pulmonary arterial smooth muscle cells

Overall, the results of the present study suggested that the expression of ZIP12 was elevated in the lung tissues and PASMCs isolated from rats with MCT-induced PAH. Overexpression of ZIP12 facilitated the proliferation and migration of Ctrl-PASMCs, and silencing ZIP12 caused the opposite effects in MCT-PAH-PASMCs. Furthermore, the results of this study also demonstrate how ZIP12 exerts pro-proliferative and pro-migratory functions that are mediated by a new mechanism in MCT-induced PAH, which is, at least partly, mediated by the AKT/ERK signaling pathways (Fig. 7). These data suggest that the development of specific pharmacological inhibitors targeting ZIP12 may be a promising intervention strategy for PAH.

## Supplementary Information

**Additional file 1: Fig. S1.** Hemodynamic measurements and morphometric analysis of pulmonary arterioles. (**A**) Representative pulmonary arterial pressure waveforms and mean pulmonary arterial pressure alteration in MCT-treated rats and control rats (n = 8 rats per group). (**B**) Assessment of Fulton index in MCT-treated rats and control rats (n = 8 rats per group). Representative images of HE staining in lung tissues (magnification, ×400) and quantification of the percentage of the vascular wall area (WA%) and the percentage of vascular wall thickness (WT%) of the pulmonary arterioles (n = 8 rats per group), the intima in MCT-treated rats was thicker and infiltration of inflammatory cells (arrowheads) (**C**), α-SMA stained pulmonary arterioles (magnification, ×400) and the proportion of muscularized pulmonary arterioles (n = 8 rats per group) (**D**), PCNA stained pulmonary arterioles (magnification, ×400) and quantification of the percentage of PCNA-positive smooth muscle cells of the pulmonary arterioles (n = 8 rats per group) (**E**), and Masson staining of pulmonary arterioles (magnification, ×400) (**F**). Abbreviations: Ctrl, control; PAH, pulmonary arterial hypertension; MCT, monocrotaline. The data are expressed as the mean ± standard deviation. *P < 0.05, **P < 0.01.

**Additional file 2: Fig. S2.** MCT treatment increased the proliferation and migration of PASMCs. Cells were serum-starved for 24 h in 0.2% FBS in DMEM-F12 and then restimulated with 10% FBS in DMEM-F12 for 48 h. (**A**) An EdU assay was performed to compare the proliferation of Ctrl-PASMCs and MCT-PAH-PASMCs (magnification, ×100; n = 7 rats per group). (**B**) A wound healing assay was performed to compare the migration of Ctrl-PASMCs and MCT-PAH-PASMCs, and the wounds were imaged every 24 h (magnification, ×40; n = 5 rats per group). (**C**, **D**) Representative Western blot and summarized data showing the protein expression of (**C**) PCNA and (**D**) cyclin D1 in Ctrl-PASMCs and MCT-PAH-PASMCs (C, n = 6 rats per group; D, n = 5 rats per group). Abbreviations: Ctrl, control; PAH, pulmonary arterial hypertension; PASMCs, pulmonary arterial smooth muscle cells; EdU, 5-ethynyl-2′deoxyuridine; MCT: monocrotaline. The data are expressed as the mean ± standard deviation. Error bars represented standard deviation. *P < 0.05, **P < 0.01.

**Additional file 3: Fig. S3.** Phosphorylation of AKT and ERK1/2 were enhanced in MCT-PAH-PASMCs upon stimulation with 10% FBS. The cells were serum-starved for 24 h in 0.2% FBS in DMEM-F12 and then restimulated with 10% FBS in DMEM-F12 for 0, 5, 15, 30, and 60 min. (**A**, **B**) Representative Western blot and summarized data showing the protein expression of (**A**) phosphorylated and total AKT and (**B**) phosphorylated and total ERK1/2 in Ctrl-PASMCs and MCT-PAH-PASMCs (A, n = 6 independent experiments; B, n = 4 independent experiments). Abbreviations: Ctrl, control; PAH, pulmonary arterial hypertension; PASMCs, pulmonary arterial smooth muscle cells; MCT, monocrotaline. The data are expressed as the mean ± standard deviation. Error bars represented standard deviation. *P < 0.05 and **P < 0.01 vs. the control group at the corresponding time point.

**Additional file 4: Fig. S4.** Transfection efficiency and the expression of ZIP12 after the transfection. (**A**) Efficiency of lentiviral infection at various MOIs for 72 h (magnification, ×100). (**B**) Transfection efficiency was determined by flow cytometry. (**C**, **D**) Protein lysates were prepared from transfected cells 72 h after the transfection, and ZIP12 protein expression levels were determined by Western blot. Representative Western blot and summarized data showing the protein expression of ZIP12 in (**C**) MCT-PAH-PASMCs and (**D**) Ctrl-PASMCs (C, n = 5 independent experiments; D, n = 3 independent experiments). Abbreviations: Ctrl, control; PAH, pulmonary arterial hypertension; PASMCs, pulmonary arterial smooth muscle cells; MOI, multiplicity of infection; MCT, monocrotaline. The data are expressed as the mean ± standard deviation. Error bars represented standard deviation. **P < 0.01.

**Additional file 5: Fig. S5.** ZIP12 overexpression promoted the proliferation and migration of Ctrl-PASMCs. Ctrl-PASMCs were infected with LV-NC or LV-ZIP12. After 72 h of infection, the cells were serum starved for 24 h in 0.2% FBS in DMEM-F12, followed by restimulation with 10% FBS in DMEM-F12 for 48 h. (**A**) The proliferation of Ctrl-PASMCs in response to ZIP12 overexpression was determined by EdU assay (magnification, ×100; n = 6 independent experiments). (**B**) The migration of Ctrl-PASMCs in response to ZIP12 overexpression was determined by wound healing assay. The wounds were imaged every 24 h (magnification, ×40; n = 5 independent experiments). (**C**, **D**) Representative Western blot and summarized data of ZIP12 overexpressing and the effect on (**C**) PCNA and (**D**) cyclin D1 protein expression (n = 4 independent experiments). Abbreviations: Ctrl, control; PASMCs, pulmonary arterial smooth muscle cells; EdU, 5-ethynyl-2′deoxyuridine. The data are expressed as the mean ± standard deviation. Error bars represented standard deviation. **P < 0.01.

**Additional file 6:** Unprocessed Western blot images.

## Data Availability

All data from this study are available in this published article.

## Abbreviations

AKT: Protein kinase B
α-SMA: α-Smooth muscle actin
AP-1: Activator protein-1
CREB: CAMP response element binding protein
CDKs: Cyclin-dependent kinases
DMEM: Dulbecco’s modified Eagle’s medium
DiO: 3,3′-Dioctadecyloxacarbocyanine perchlorate
DUSPs: Dual‐specificity phosphatases
DAPI: 4′,6-Diamidino-2-phenylindole
ERK: Extracellular regulated protein kinases
ED: External diameter
ECL: Enhanced chemiluminescence
EdU: 5-Ethynyl-2′-deoxyuridine
EGFR: Epidermal growth factor receptor
FBS: Fetal bovine serum
GSK-3β: Glycogen synthase kinase-3β
HE: Hematoxylin eosin
IR: Insulin receptor
IGF-1R: Insulin-like growth factor 1 receptor
LA: Lumen area
MCT: Monocrotaline
MOI: Multiplicity of infection
MAPK: Mitogen-activated protein kinase
mTOR: Mammalian target of rapamycin
PAH: Pulmonary arterial hypertension
PASMCs: Pulmonary arterial smooth muscle cells
PI3K: Phosphoinositide 3-kinases
PCNA: Proliferating cell nuclear antigen
PMSF: Phenylmethanesulphonylfluoride
PVDF: Polyvinylidene fluoride
PTPs: Protein tyrosine phosphatases
PP2A: Protein phosphatase 2A
PTEN: Phosphatase and tensin homolog
PKC: Protein kinase C
RVHI: Right ventricular hypertrophy index
RAC1: Ras-related C3 botulinum toxin substrate 1
SDS-PAGE: Sodium dodecyl sulfate–polyacrylamide gel electrophoresis
shRNA: Short hairpin RNA
TA: Total area
TBST: Tris-buffered saline with Tween 20
WT: Wall thickness
WT%: The percentage of vascular wall thickness
WA%: The percentage of the vascular wall area
ZIP: Zinc IRT-like protein
ZnT: Zinc transporter
