# Supplementary figures and images for: The zinc transporter ZIP12 regulates monocrotaline-induced proliferation and migration of pulmonary arterial smooth muscle cells via the AKT/ERK signaling pathways
Source: BMC Pulm Med. 2022 Mar 28;22:111. doi: 10.1186/s12890-022-01905-3 (PMC8962172; doi:10.1186/s12890-022-01905-3)

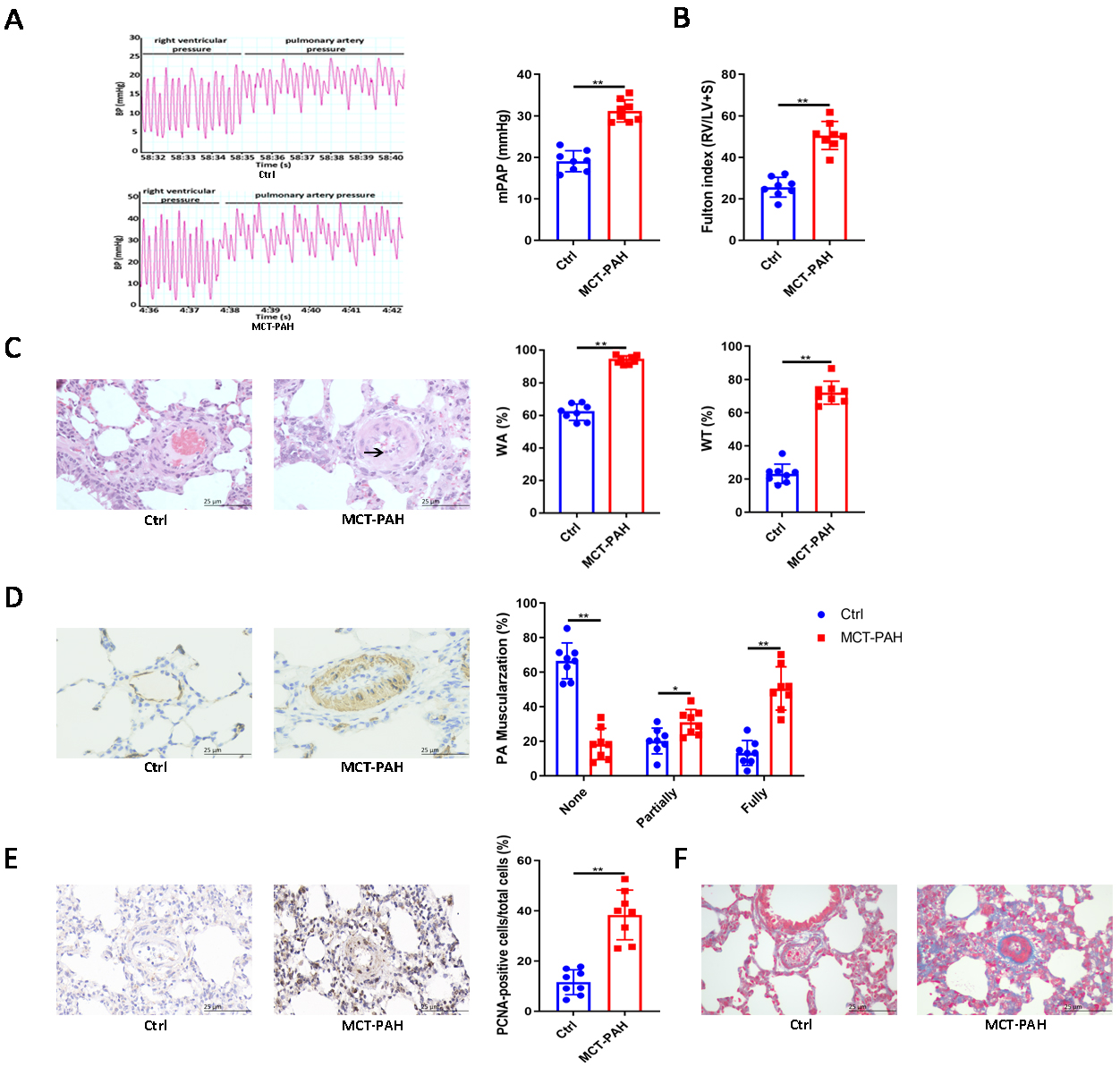

Supplement: Supplementary file 1 — Additional file 1: Fig. S1. Hemodynamic measurements and morphometric analysis of pulmonary arterioles. (A) Representative pulmonary arterial pressure waveforms and mean pulmonary arterial pressure alteration in MCT-treated rats and control rats (n = 8 rats per group). (B) Assessment of Fulton index in MCT-treated rats and control rats (n = 8 rats per group). Representative images of HE staining in lung tissues (magnification, ×400) and quantification of the percentage of the vascular wall area (WA%) and the percentage of vascular wall thickness (WT%) of the pulmonary arterioles (n = 8 rats per group), the intima in MCT-treated rats was thicker and infiltration of inflammatory cells (arrowheads) (C), α-SMA stained pulmonary arterioles (magnification, ×400) and the proportion of muscularized pulmonary arterioles (n = 8 rats per group) (D), PCNA stained pulmonary arterioles (magnification, ×400) and quantification of the percentage of PCNA-positive smooth muscle cells of the pulmonary arterioles (n = 8 rats per group) (E), and Masson staining of pulmonary arterioles (magnification, ×400) (F). Abbreviations: Ctrl, control; PAH, pulmonary arterial hypertension; MCT, monocrotaline. The data are expressed as the mean ± standard deviation. *P < 0.05, **P < 0.01. [file 12890_2022_1905_MOESM1_ESM.jpg]

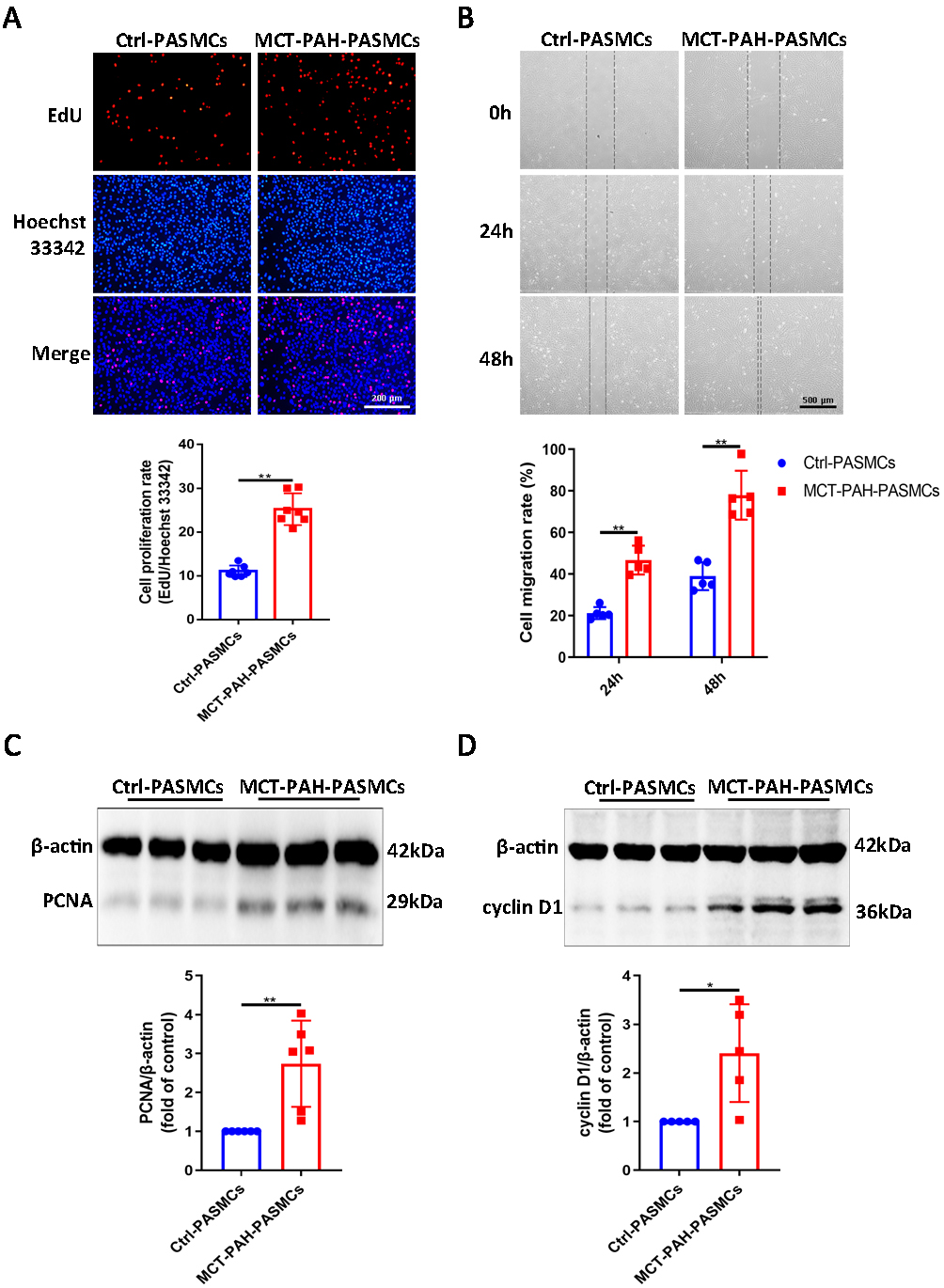

Supplement: Supplementary file 2 — Additional file 2: Fig. S2. MCT treatment increased the proliferation and migration of PASMCs. Cells were serum-starved for 24 h in 0.2% FBS in DMEM-F12 and then restimulated with 10% FBS in DMEM-F12 for 48 h. (A) An EdU assay was performed to compare the proliferation of Ctrl-PASMCs and MCT-PAH-PASMCs (magnification, ×100; n = 7 rats per group). (B) A wound healing assay was performed to compare the migration of Ctrl-PASMCs and MCT-PAH-PASMCs, and the wounds were imaged every 24 h (magnification, ×40; n = 5 rats per group). (C, D) Representative Western blot and summarized data showing the protein expression of (C) PCNA and (D) cyclin D1 in Ctrl-PASMCs and MCT-PAH-PASMCs (C, n = 6 rats per group; D, n = 5 rats per group). Abbreviations: Ctrl, control; PAH, pulmonary arterial hypertension; PASMCs, pulmonary arterial smooth muscle cells; EdU, 5-ethynyl-2′deoxyuridine; MCT: monocrotaline. The data are expressed as the mean ± standard deviation. Error bars represented standard deviation. *P < 0.05, **P < 0.01. [file 12890_2022_1905_MOESM2_ESM.jpg]

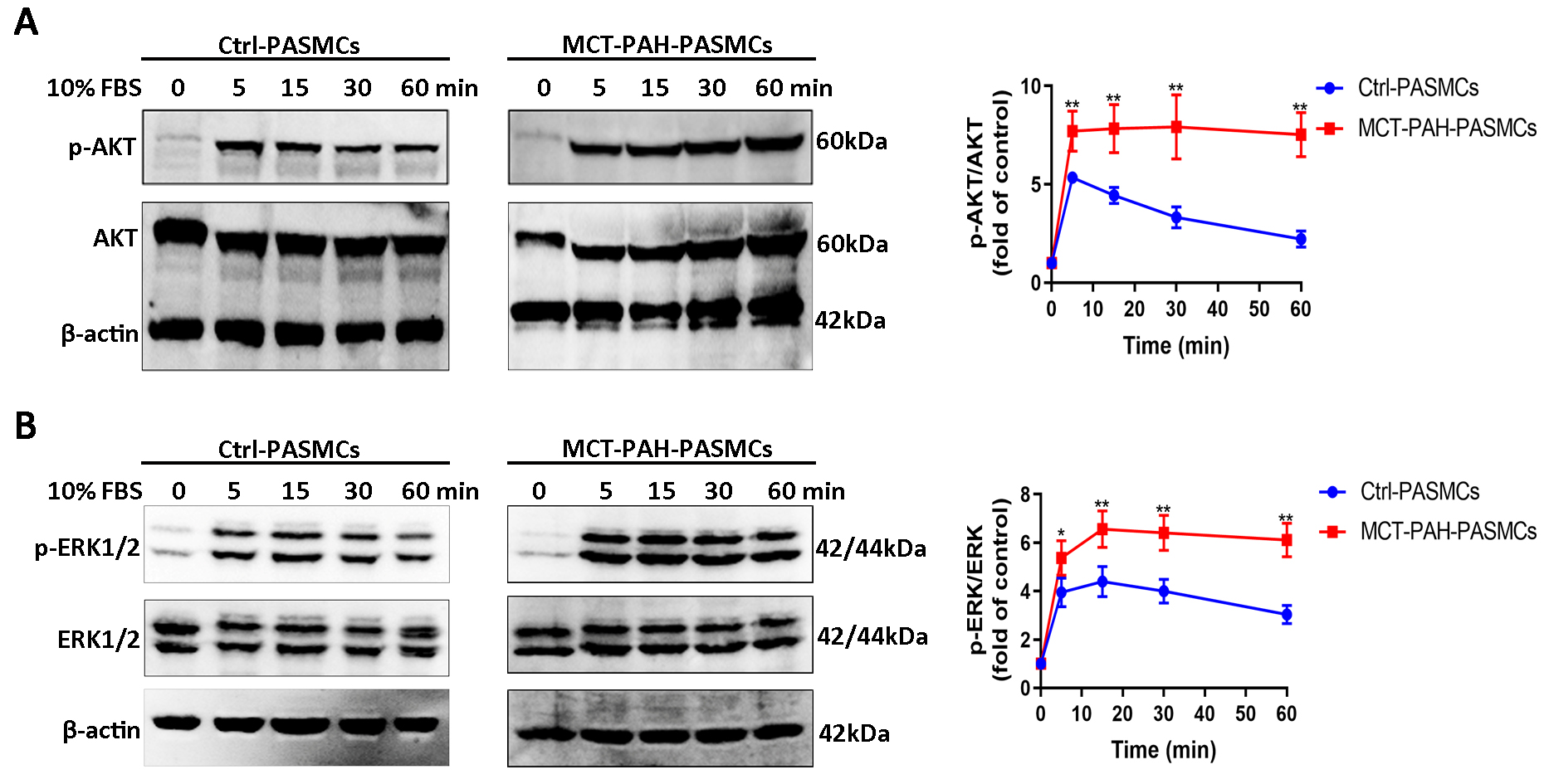

Supplement: Supplementary file 3 — Additional file 3: Fig. S3. Phosphorylation of AKT and ERK1/2 were enhanced in MCT-PAH-PASMCs upon stimulation with 10% FBS. The cells were serum-starved for 24 h in 0.2% FBS in DMEM-F12 and then restimulated with 10% FBS in DMEM-F12 for 0, 5, 15, 30, and 60 min. (A, B) Representative Western blot and summarized data showing the protein expression of (A) phosphorylated and total AKT and (B) phosphorylated and total ERK1/2 in Ctrl-PASMCs and MCT-PAH-PASMCs (A, n = 6 independent experiments; B, n = 4 independent experiments). Abbreviations: Ctrl, control; PAH, pulmonary arterial hypertension; PASMCs, pulmonary arterial smooth muscle cells; MCT, monocrotaline. The data are expressed as the mean ± standard deviation. Error bars represented standard deviation. *P < 0.05 and **P < 0.01 vs. the control group at the corresponding time point. [file 12890_2022_1905_MOESM3_ESM.jpg]

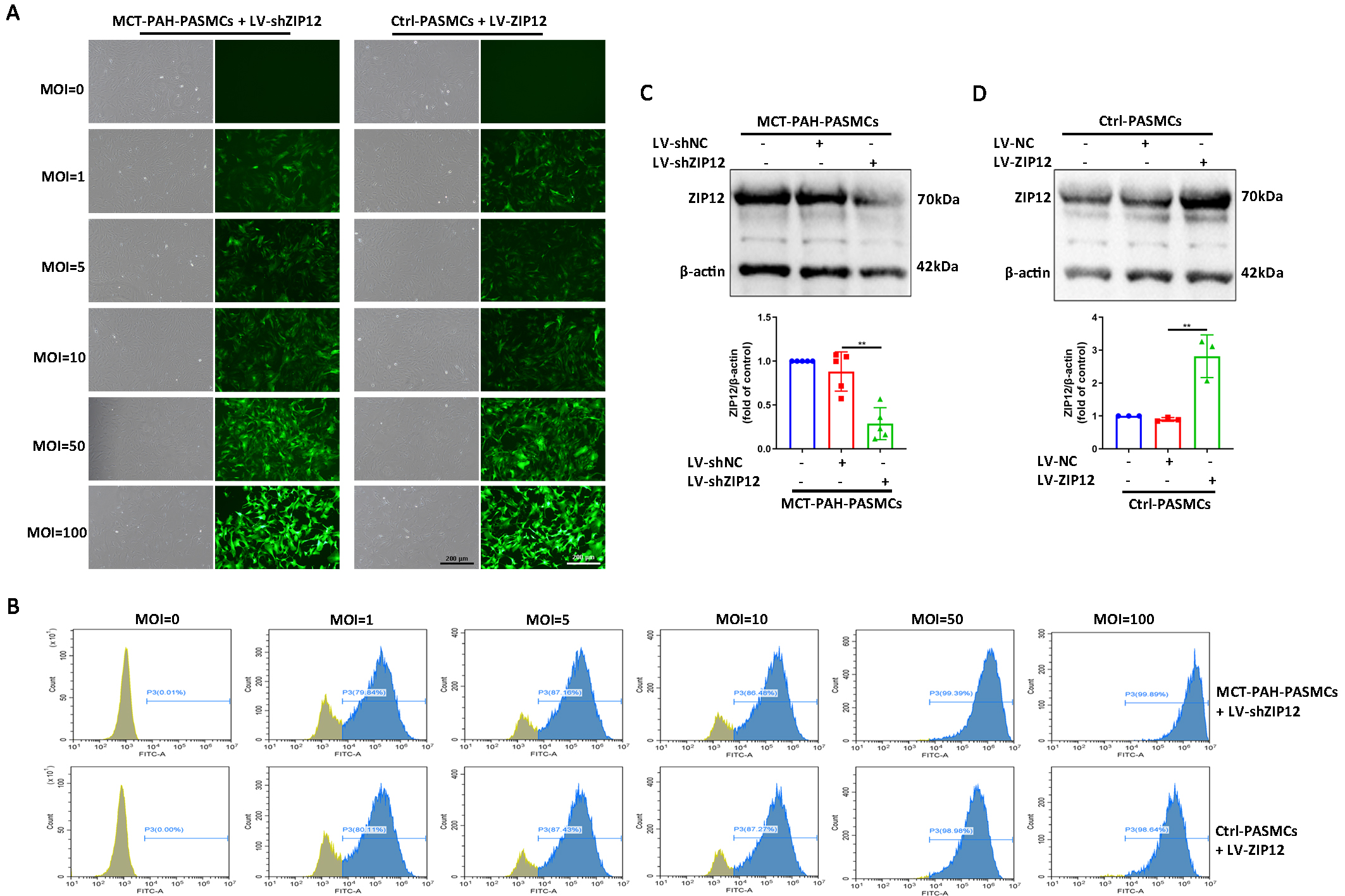

Supplement: Supplementary file 4 — Additional file 4: Fig. S4. Transfection efficiency and the expression of ZIP12 after the transfection. (A) Efficiency of lentiviral infection at various MOIs for 72 h (magnification, ×100). (B) Transfection efficiency was determined by flow cytometry. (C, D) Protein lysates were prepared from transfected cells 72 h after the transfection, and ZIP12 protein expression levels were determined by Western blot. Representative Western blot and summarized data showing the protein expression of ZIP12 in (C) MCT-PAH-PASMCs and (D) Ctrl-PASMCs (C, n = 5 independent experiments; D, n = 3 independent experiments). Abbreviations: Ctrl, control; PAH, pulmonary arterial hypertension; PASMCs, pulmonary arterial smooth muscle cells; MOI, multiplicity of infection; MCT, monocrotaline. The data are expressed as the mean ± standard deviation. Error bars represented standard deviation. **P < 0.01. [file 12890_2022_1905_MOESM4_ESM.jpg]

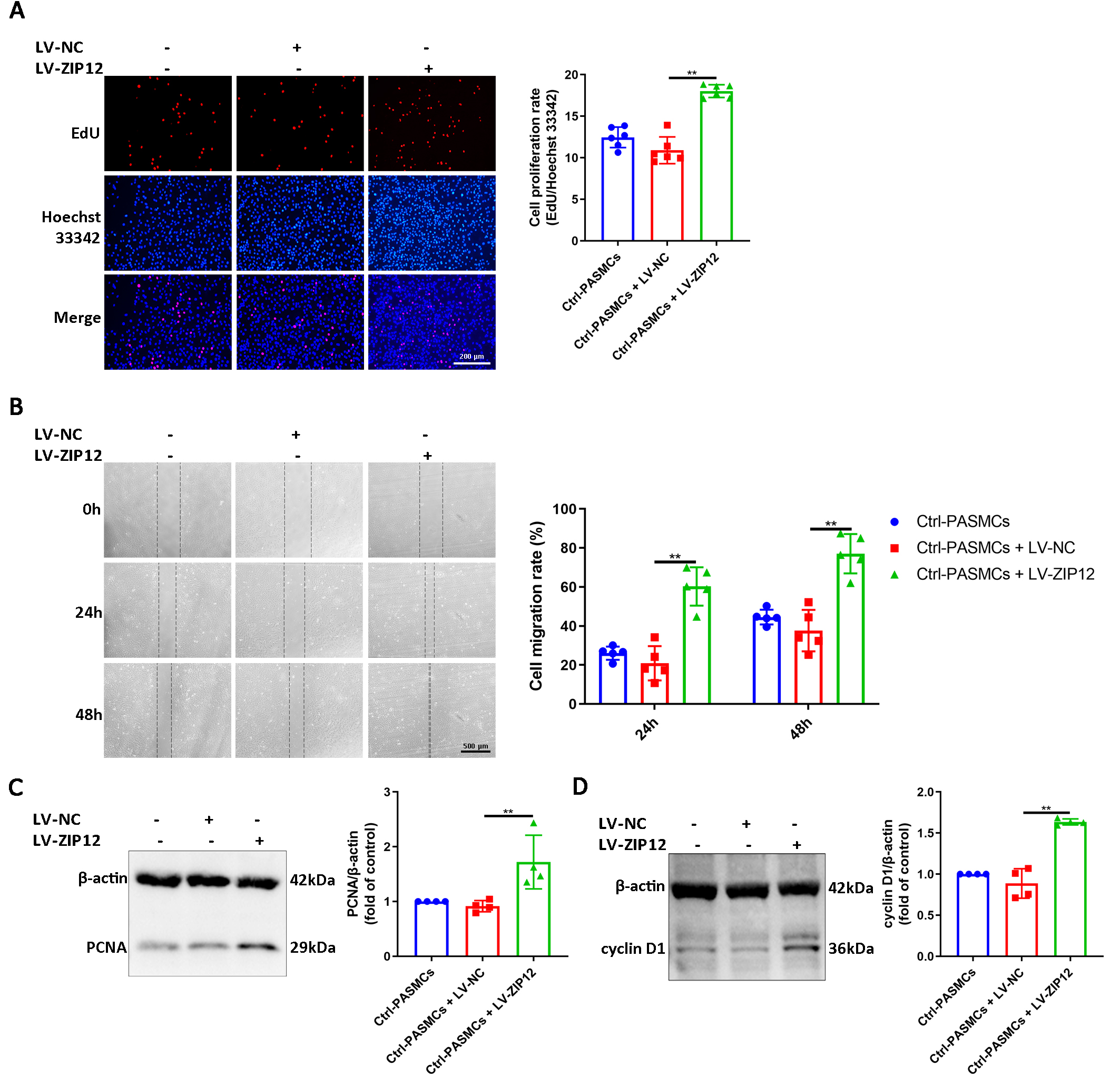

Supplement: Supplementary file 5 — Additional file 5: Fig. S5. ZIP12 overexpression promoted the proliferation and migration of Ctrl-PASMCs. Ctrl-PASMCs were infected with LV-NC or LV-ZIP12. After 72 h of infection, the cells were serum starved for 24 h in 0.2% FBS in DMEM-F12, followed by restimulation with 10% FBS in DMEM-F12 for 48 h. (A) The proliferation of Ctrl-PASMCs in response to ZIP12 overexpression was determined by EdU assay (magnification, ×100; n = 6 independent experiments). (B) The migration of Ctrl-PASMCs in response to ZIP12 overexpression was determined by wound healing assay. The wounds were imaged every 24 h (magnification, ×40; n = 5 independent experiments). (C, D) Representative Western blot and summarized data of ZIP12 overexpressing and the effect on (C) PCNA and (D) cyclin D1 protein expression (n = 4 independent experiments). Abbreviations: Ctrl, control; PASMCs, pulmonary arterial smooth muscle cells; EdU, 5-ethynyl-2′deoxyuridine. The data are expressed as the mean ± standard deviation. Error bars represented standard deviation. **P < 0.01. [file 12890_2022_1905_MOESM5_ESM.jpg]
