# Supplementary material for: The zinc transporter ZIP12 regulates monocrotaline-induced proliferation and migration of pulmonary arterial smooth muscle cells via the AKT/ERK signaling pathways
Source: BMC Pulm Med. 2022 Mar 28;22:111. doi: 10.1186/s12890-022-01905-3 (PMC8962172; doi:10.1186/s12890-022-01905-3)

Western Blot images

Figure 1D

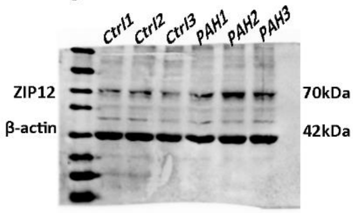

Figure 1E

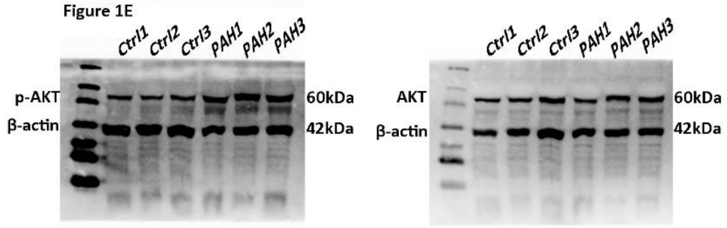

Figure 1F

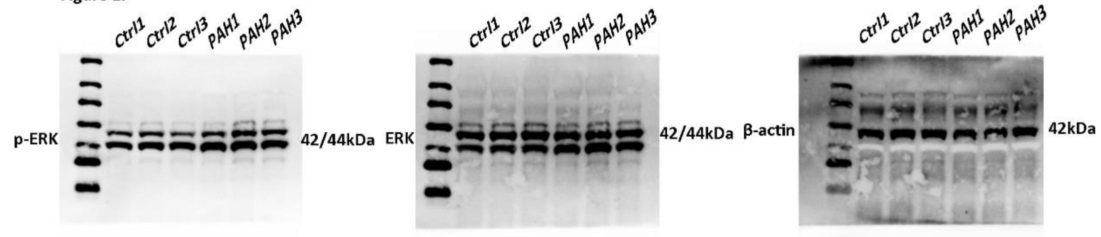

Figure 1G

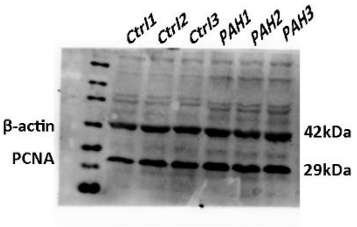

Figure 1H

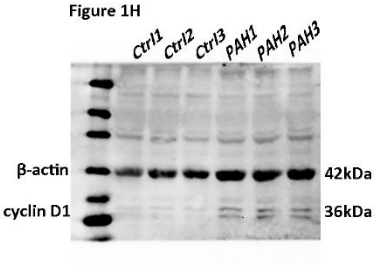

Figure 2C

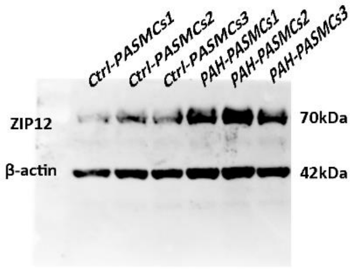

Figure 3C

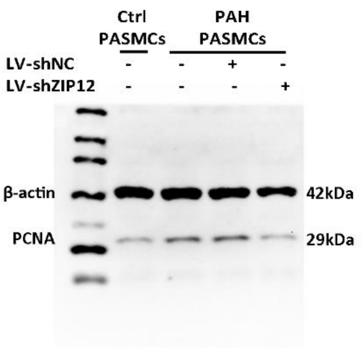

Figure 3D

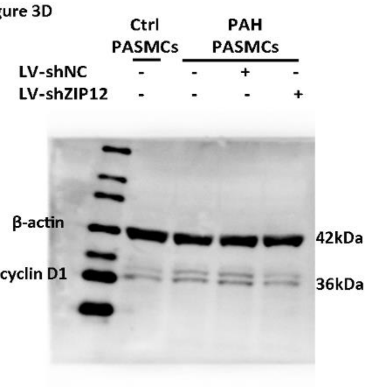

Figure 4A

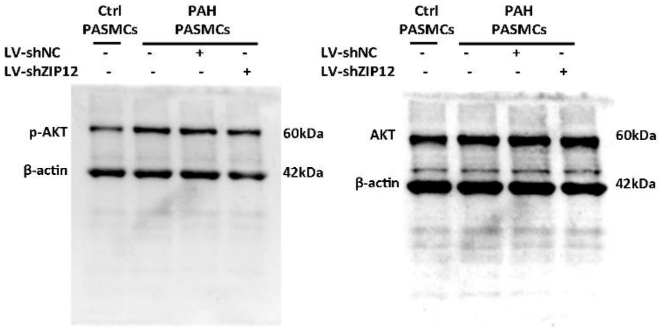

Figure 4B

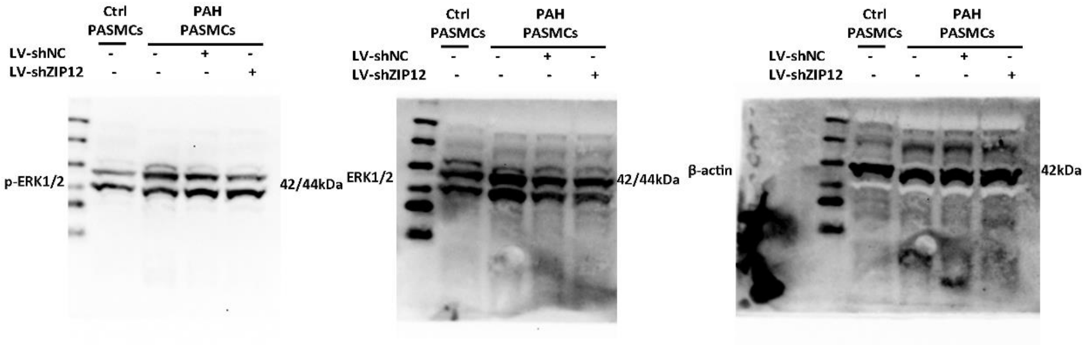

Figure 5A

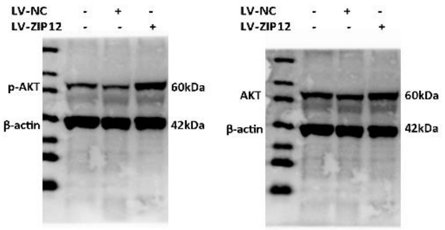

Figure 5B

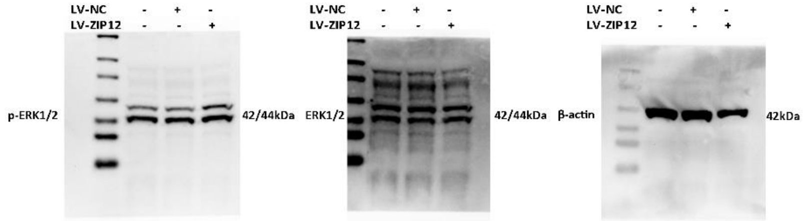

Figure 5C

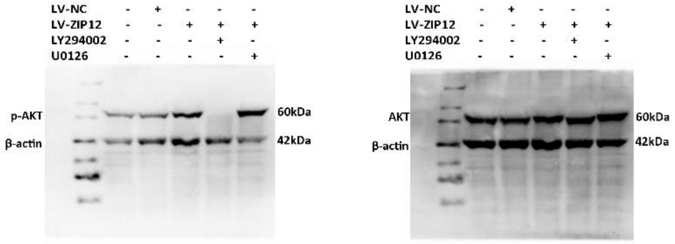

Figure 5D

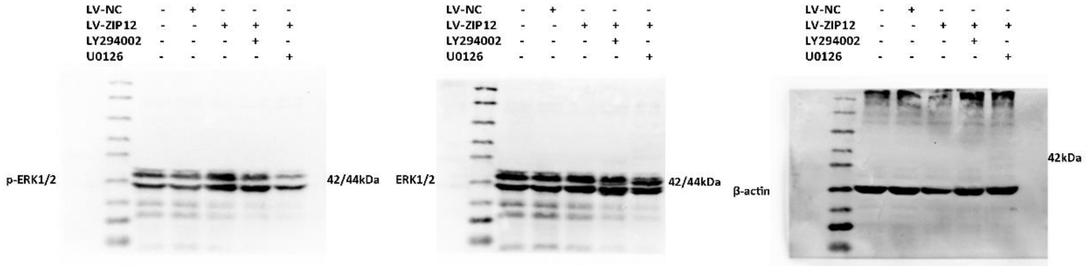

Figure 6C

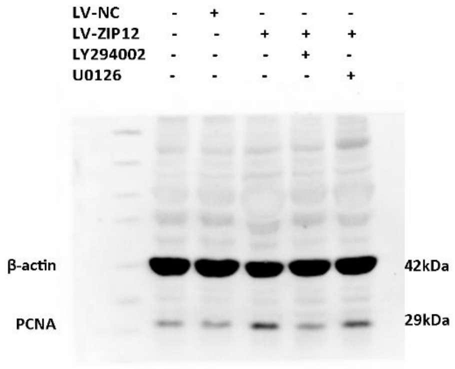

Figure 6D

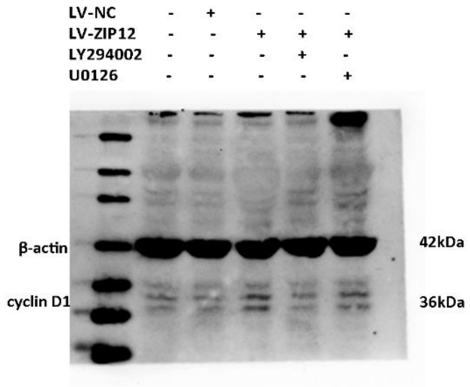

Supplementary Figure 2C

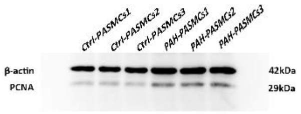

Supplementary Figure 2D

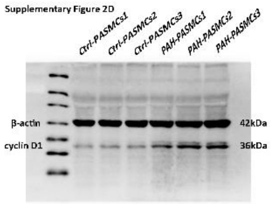

Supplementary Figure 3A

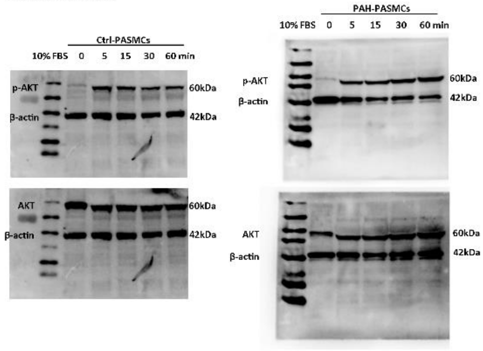

Supplementary Figure 3B

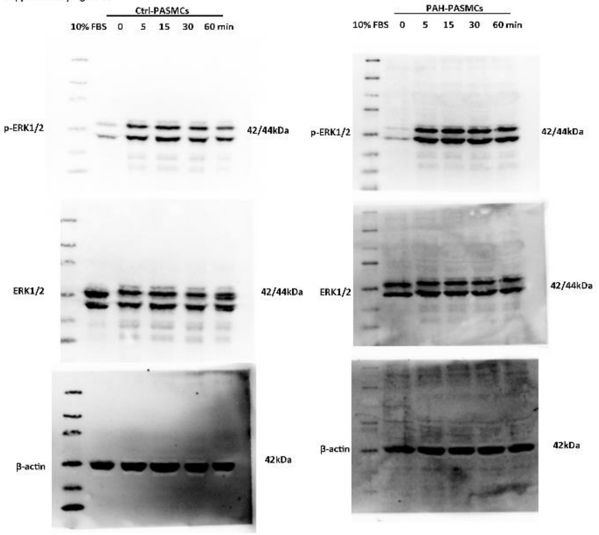

Supplementary Figure 4C

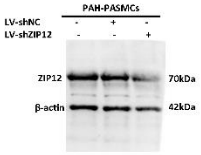

Supplementary Figure 4D

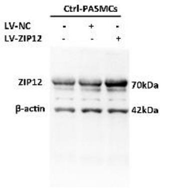

Supplementary Figure 5C

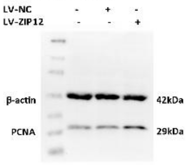

Supplementary Figure 5D

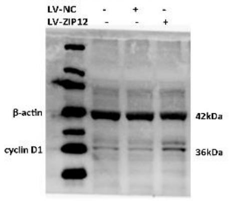

Supplement: Supplementary file 6 — Additional file 6: Unprocessed Western blot images. [file 12890_2022_1905_MOESM6_ESM.pdf]
